# A mutation in *VPS15* (*PIK3R4)* causes a ciliopathy and affects IFT20 release from the *cis*-Golgi

**DOI:** 10.1038/ncomms13586

**Published:** 2016-11-24

**Authors:** Corinne Stoetzel, Séverine Bär, Johan-Owen De Craene, Sophie Scheidecker, Christelle Etard, Johana Chicher, Jennifer R. Reck, Isabelle Perrault, Véronique Geoffroy, Kirsley Chennen, Uwe Strähle, Philippe Hammann, Sylvie Friant, Hélène Dollfus

**Affiliations:** 1 Medical Genetics Laboratory, INSERM U1112, Institute of Medical Genetics of Alsace, University of Strasbourg, Strasbourg Medical School, 67000 Strasbourg, France; 2 Department of Molecular and Cellular Genetics, UMR7156, Centre National de Recherche Scientifique (CNRS), Université de Strasbourg, 67084 Strasbourg, France; 3 Institut für Toxikologie und Genetik, Campus Nord, Karlsruher Institut für Technologie, Hermann-von-Helmholtz-Platz 1, 76344 Eggenstein Leopoldshafen, Germany; 4 Institut de Biologie Moléculaire et Cellulaire (IBMC), Plateforme Protéomique Strasbourg—Esplanade, CNRS FRC1589, 67084 Strasbourg, France; 5 Laboratory of Genetics in Ophthalmology, INSERM UMR1163, Institut Imagine, Université Paris Descartes Sorbonne Paris Cité, Hôpital Necker, 75015 Paris, France; 6 Centre de Référence pour les affections rares en génétique ophtalmologique, CARGO, Filière SENSGENE, Hôpitaux Universitaires de Strasbourg, 67091 Strasbourg, France

## Abstract

Ciliopathies are a group of diseases that affect kidney and retina among other organs. Here, we identify a missense mutation in *PIK3R4* (phosphoinositide 3-kinase regulatory subunit 4, named *VPS15*) in a family with a ciliopathy phenotype. Besides being required for trafficking and autophagy, we show that VPS15 regulates primary cilium length in human fibroblasts, as well as ciliary processes in zebrafish. Furthermore, we demonstrate its interaction with the golgin GM130 and its localization to the Golgi. The VPS15-R998Q patient mutation impairs Golgi trafficking functions in humanized yeast cells. Moreover, in VPS15-R998Q patient fibroblasts, the intraflagellar transport protein IFT20 is not localized to vesicles trafficking to the cilium but is restricted to the Golgi. Our findings suggest that at the Golgi, VPS15 and GM130 form a protein complex devoid of VPS34 to ensure the IFT20-dependent sorting and transport of membrane proteins from the *cis*-Golgi to the primary cilium.

## Abstract (web summary)

VPS15 is known as a VPS34-associated protein that functions in intracellular trafficking and autophagy. Here the authors identify a role for VPS15 in ciliopathy and ciliary phenotypes, and show that it interacts with GM130 and functions in IFT20-dependent *cis*-Golgi to cilium trafficking.

## Graphical abstract

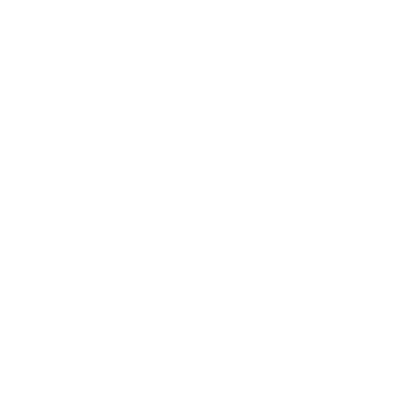

The primary cilium is a non-motile polarized microtubule-based hair-like structure protruding from the surface of many eukaryotic cells, with the exception of higher plants and fungi. Primary cilia are sensing extracellular molecules and environmental stimuli. By dynamic exchanges of signalling molecules, they convert extracellular signals to intracellular responses^1^. They have a pivotal role in different signalling cascades: the Sonic Hedgehog, Wnt, Notch signalling and mTOR pathways. In humans, cilia dysfunctions can result in developmental defects such as *situs inversus*, polydactyly or central nervous system abnormalities as well as progressive organ alteration, including, retinal degeneration or cystic kidneys and form a class of clinically and genetically heterogeneous diseases termed ciliopathies^2^,^3^. Ciliopathies are due to disease-causing mutations in genes encoding ciliary proteins and their study has revealed various actors of ciliary biogenesis and function, as well as unexpected links to previously characterized cellular pathways^2^.

Early stages of ciliogenesis are characterized by the maturation of the mother centriole into a basal body as it migrates from its perinuclear position to the plasma membrane, followed by cilium elongation after extension of the axoneme^4^. Axoneme extension and cilia signalling largely rely on the evolutionary conserved mechanism of intraflagellar transport (IFT) via the IFT-A/B protein complexes that ensure bidirectional trafficking along the ciliary axoneme, by using kinesin-2 (anterograde) and dynein (retrograde) molecular motors. Among these IFTs, only the IFT20 protein has a dual localization at Golgi and basal body. At the Golgi, IFT20 ensures the sorting of ciliary cargo from the *cis*-Golgi to the base of the cilium^5^,^6^. Ciliary membrane proteins are either delivered from the Golgi to the plasma membrane before moving towards the basal body or they are directly transported from the Golgi to the cilium^4^. At the base of the cilium, a complex termed BBSome formed by a subset of BBS proteins recognizes ciliary cargoes^7^,^8^. The BBSome shares structural features with clathrin and coatomer coats (COPI and COPII) forming a coat on liposome membranes once recruited by Arl6-GTP^7^,^8^.

Here, we report the identification of a mutation in the *PIK3R4/VPS15* gene (MIM 602610, NM_014602.2: c.2993G>A, R998Q) in three affected siblings with a ciliopathy phenotype. VPS15 (Vacuolar Protein Sorting 15) encodes the phosphoinositide 3-kinase regulatory subunit 4 required for the synthesis of the lipid phosphatidylinositol 3-phosphate (PtdIns3P). VPS15 was first identified and characterized in yeast *Saccharomyces cerevisiae* after its isolation in different genetic screens performed to identify proteins required for vacuolar protein sorting (VPS)^9^. VPS15 binds to and regulates the class III PtdIns3P lipid kinase VPS34/PIK3C3 converting PtdIns to PtdIns3P. VPS15 in association with VPS34 is involved in two well-studied protein complexes conserved from yeast to mammals, the UVRAG/Beclin1 and Atg14L/Beclin1 complex required for membrane trafficking and autophagy, respectively^10^,^11^. In metazoan, *VPS15* is an essential gene since drosophila homozygous deletion mutant and whole-body *Vps15*-deficient mice (*Vps15*^*−/−*^) display early L3 larval stage or early-embryonic lethality a phenotype similar to the one observed for *Vps34*^*−/−*^ mice^11^,^12^. The VPS15 protein is composed of an amino (N)-terminal protein kinase, a HEAT repeat, a coiled coil and seven carboxy (C)-terminal WD40 domains.

In the present work, we describe for the first time patients with a ciliopathy phenotype carrying a mutation in *VPS15* as well as the link between VPS15 and cilia trafficking by using three model organisms (human, zebrafish and yeast). We show that the VPS15-R998Q patient mutation affects the primary cilium length in human fibroblasts, induces axial curvature and kidney cysts in zebrafish embryos and impairs the dominant-negative Golgi to vacuole trafficking phenotype of hVPS15 in yeast cells. We also show that in human fibroblasts a pool of VPS15 localizes to the Golgi and interacts with the golgin GM130 ensuring efficient loading of IFT20 in vesicles addressed from the *cis*-Golgi to the basal body of the cilium. This work suggests that VPS15 and GM130 function together at the Golgi to ensure the IFT20-dependent sorting and transport of proteins addressed to the ciliary membrane.

## Results

### A mutation in the *VPS15* gene in a family with a ciliopathy

**Figure 1:**
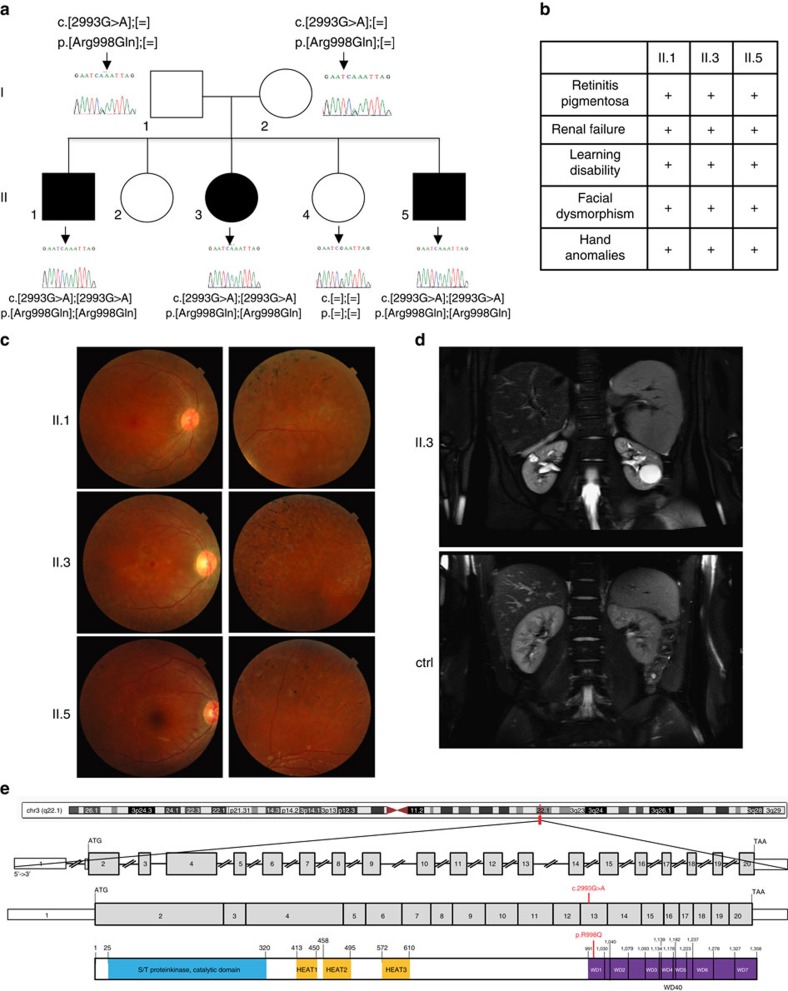
Pedigree and clinical presentation of the family affected by VPS15-R998Q mutation. (**a**) Pedigree of the family with affected members in black and results of Sanger sequencing confirming co-segregation of the mutation with the disease. (**b**) Main clinical features of II.1, II.3 and II.5 affected individuals. (**c**) Fundus photographs (respectively right eye, left eye) of II.3 and II.5 individuals showing on the left sides a central view of the retina with the posterior pole of each right eye of the patients with mild narrowing of the retinal vasculature and moderate visible changes at the macula (best seen for II.3). On the right sides, peripheral views show high level of pigment epithelium heterogeneity/atrophy with pigment migrations typical for retinitis pigmentosa (arrows). (**d**) Abdominal MRI (SPAIR fat-suppression technique, Achieva PHILIPS 3 T) of II.3 and of a normal individual (Ctrl) for comparison (long and short axis are drawn on the normal kidney view). Coronal view of the kidneys showing the very reduced length of patient II.3 kidneys, the right and left kidneys being 9.1 and 8.2 cm on long axis and 4.3 and 4.1 cm on short axis, compared with the mean 11 cm (long axis) and 5 cm (short axis) length for the normal kidneys. The MRI also shows corticomedullary microcysts as well as larger cysts (arrows), the one in the left kidney with 3 cm in diameter. (**e**) Schematic representation of the gene, mRNA and VPS15 protein with the different protein domains and the location of the mutation indicated in red.

Three siblings (patients II.1, II.3 and II.5 in Fig. 1a) born from Algerian parents (originating from the same village and with high probability of distant consanguinity) were referred for early-onset retinal degeneration and progressive renal disease (CARGO, Rare Eye Diseases Center, Strasbourg University Hospital; Fig. 1b). The proband (II.1) was reported with night blindness as well as progressive visual impairment since the age of 5. An unrecordable electroretinogram at age 11 confirmed the diagnosis of retinitis pigmentosa. Fundus examinations revealed progressive widespread retinal dystrophy associated with pigment migration (Fig. 1c). He also developed progressive renal failure with glomerular and tubular dysfunctions followed by progressive kidney atrophy reminiscent of nephronophthysis requiring dialysis at age 24. General clinical examination showed a small stature (−3 s.d.) due to growth hormone deficiency, overweight (BMI 28 kg m^−2^), prognathism (similar to his father suggesting an independent dominant trait), enlarged hands with brachydactyly and clinodactyly of the fifth digit, and mild learning difficulties (Fig. 1b). The younger sister (II.3) at age 6 presented growth delay (−2 s.d.) and was diagnosed with retinitis pigmentosa. Besides her history of brachial plexus palsy at birth and dysmenorrhea at age 19, she presented the same physical features as her brother (prognathism, enlarged hands, bradymetacarpy and brachymesophalangy, and clinodactyly of the fifth finger). She was diagnosed with kidney manifestations at age 14 and had moderate renal dysfunction at the age 19. Abdominal magnetic resonance imaging (MRI) revealed reduced size kidneys (9 cm axial length for the right and 8 cm for the left; normal axial length 12 cm) filled with multiple corticomedullary microcysts and a larger cyst (3 cm) in the left kidney (Fig. 1d). The kidney biopsy showed a diffuse tubulo-interstitial nephropathy. The youngest sibling (II.5) had a history of congenital hypotonia, brachial plexus palsy at birth, language delay and mild learning difficulties (Fig. 1b). Fundus examination performed at age 4 revealed retinitis pigmentosa, confirmed by an unrecordable electroretinogram. General clinical examination revealed the same gestalt as probands II.1 and II.3. Proteinuria appeared at age 4 and he is now developing a progressive nephropathy. Thus, three out of five siblings of this family exhibited typical ciliopathy features of retinitis pigmentosa, renal dysfunction and developmental anomalies prompting for molecular investigations to identify the causal mutation.

To identify the genetic mutation responsible for the disease, we screened a panel of 30 ciliopathy-related genes and we found no mutations. The genome-wide homozygosity mapping combined to whole-exome sequencing of the three affected siblings (II.1, II.3, II.5) and the healthy sister (II.4) revealed only four homozygous regions specific to the three affected individuals: two on chromosome 3 and one on chromosomes 6 and 20 (Supplementary Fig. 1). Sequencing data processing and variant calling (SNP and InDels) with the Genome Analysis Toolkit revealed 60,612 to 63,887 genetic variants per proband (Supplementary Table 1). Variant filtering using stringent criteria reduced the number of genetics variants to respectively 1,085, 793, 1,124 and 2,009 variants per proband (Supplementary Table 1). To identify variants consistent with autosomal recessive disease inheritance, we kept only compound heterozygous or homozygous variants, reducing the number of variants to two compound heterozygous variants (in the *AK9* and *RECQL4*/*MFSD3* genes) and one homozygous variant (in the *PIK3R4* gene). Using manual curation based on the known biological, physiological or functional relevance to the disease of the candidate genes, we were left with a single homozygous variant in the *PIK3R4* gene, named herein *VPS15*: p.Arg998Gln, c.2993G>A, Chr3:(GRCh37):g.130422672C>T. The variant and its cosegregation with the phenotype in the family was confirmed by Sanger sequencing. Overall, deleterious or potentially pathogenic homozygous or compound heterozygous variants were not found in 382 patients with presumed ciliopathy and yet unknown molecular origin (our databases and the databases of three laboratories specialized in ciliopathies and other rare genetic diseases) and in 7,360 patients screened for other rare diseases (Institut IMAGINE). The PolyPhen-2 (polymorphism phenotyping v2) software, a tool analysing impact of amino acid substitutions on protein structure and function^13^, predicts that this R998Q substitution is ‘probably damaging' with a HumVar score 0.994. The residue Arg998 is in the first WD40 repeat of the VPS15 protein (Fig. 1e), a domain known to be important for protein–protein interactions. Indeed, the WD40 domain of the yeast Vps15 protein is sufficient to bind Atg14 (ref. 14), bridges the Vps38/Atg6 heterodimer to form the Vps15-Vps34 complex II (ref. 15) and in human cells the NRBF2 protein interacts with the VPS15 WD40 domain^16^.

### Cilia are shorter in VPS15-R998Q patient fibroblasts

**Figure 2:**
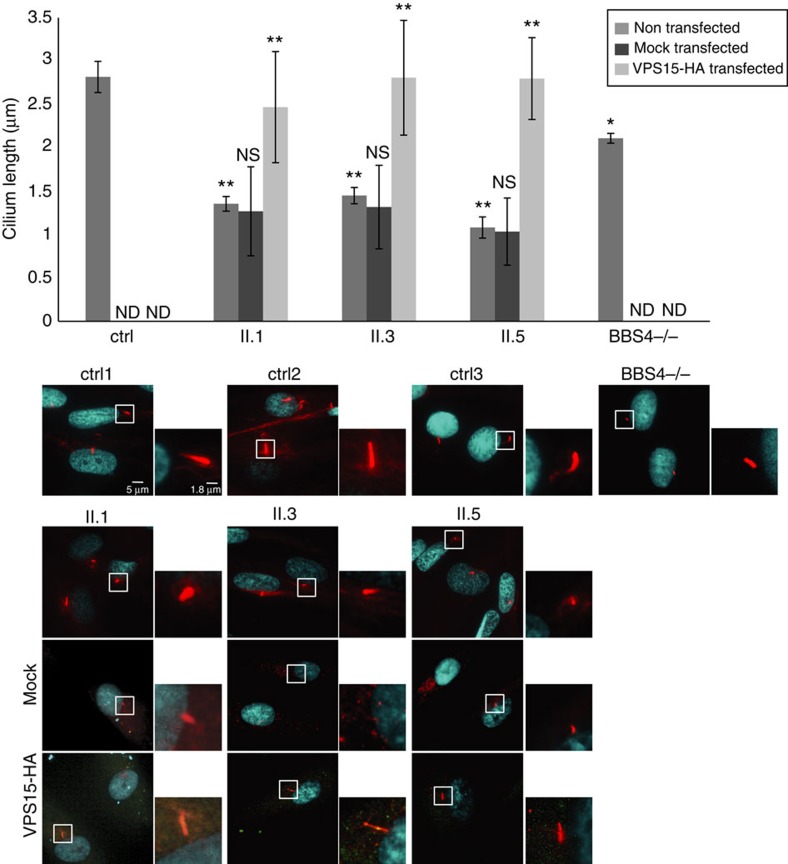
Patient fibroblasts have shorter primary cilia than control cells and this can be rescued by expression of wild-type VPS15. Patient (II.1, II.3, II.5 and *BBS4*^−/−^) and control (ctrl1, ctrl2, ctrl3) skin fibroblasts were transfected or not with VPS15-HA, serum deprived for 24 h, fixed and stained with DAPI (cyan) and acetylated α-tubulin antibodies (red). Length of cilia was measured using the ImageJ analysis program and mean of 20–200 measurements determined for each experiment. The data shown are the mean of three independent experiments and error bars represent s.d. The control Ctrl is the mean of ctrl1, 2 and 3. Statistical significance was determined using the Student's *t*-test, *P<0.05, **P<0.005. Significance is determined relative to ctrl for non-transfected cells and relative to mock transfected cells for VPS15-HA transfected cells. ND, not done; NS, non-significant.

Some ciliopathies present with impaired ciliogenesis or shorter cilia, while others are characterized by longer cilia. To investigate whether the symptoms observed in patients were associated with a ciliary phenotype, skin fibroblasts from patients II.1, II.3 and II.5 and age-matched control fibroblasts were grown to confluence in medium containing 10% fetal calf serum (FCS), then washed and starved by serum deprivation for 24 h (−FCS) to induce growth arrest and cilium formation. Primary cilia were labelled with an antibody directed against acetylated α-tubulin highlighting the axoneme (Fig. 2). Percentage of ciliated cells was similar in control (80%) and patient cells (73%). However, measuring cilium length in the age-matched healthy controls and in the patient fibroblasts showed that in cells from patients II.1, II.3 and II.5, the cilia were about 50% shorter than in the corresponding control cells (Fig. 2). Fibroblasts from a patient carrying a homozygous *BBS4* deletion were used as positive control^17^. As expected, the cilium length of *BBS4*^*−/−*^ cells was reduced, however to a lesser extent compared with the VPS15-R998Q patient cells. This result shows that skin fibroblasts from patients carrying the VPS15-R998Q mutation present a ciliary phenotype characterized by shorter primary cilia. To confirm that this phenotype is due to the missense mutation in VPS15, skin fibroblasts from patients were transfected with a plasmid expressing 3xHA tagged VPS15 cDNA. The proper expression of VPS15-HA from this plasmid was checked by western blot after transfection of the retinal pigment epithelial cell line hTERT-RPE1 (Supplementary Fig. 2A), then by immunofluorescence in control fibroblasts grown in complete medium or serum-deprived to confirm the Golgi localization (Supplementary Fig. 2B).

Finally, the patient fibroblasts were either mock transfected or transfected with the VPS15-HA construct 6 h before serum deprivation. Twenty-four hours post serum deprivation, the cells were fixed and stained as described above and the cilium length determined. The VPS15-HA protein was able to rescue the short-cilium phenotype in all the three patients confirming that the VPS15-R998Q mutation is responsible for this defect. Interestingly, while transfection with VPS15-HA and subsequent serum deprivation was deleterious to all the cells and resulted in about 70% mortality, the surviving cells appeared to be those with the lower expression of VPS15-HA, suggesting a negative effect of VPS15 overexpression.

### VPS15-RQ associated with ciliopathy phenotypes in zebrafish

Zebrafish *Danio rerio* is a well-established model for human ciliopathies^18^. Antisense morpholino injection was used to elucidate a putative ciliary function of Vps15 in zebrafish by searching for ciliopathy-related phenotypes (curvature of body axis and kidney cysts). The zebrafish z*vps15/pik3r4* gene (ENSDARG0000000469) maps to LG16 and has two predicted splice variants encoding two putative proteins of 1,340 and 1,347 amino acids. Sequencing and BLAST analysis indicated that only the shorter variant was present (data not shown), encoding a protein with a high degree of sequence similarity to mammalian VPS15 (82% identity to hVPS15) with conservation of the R998 residue at position 976 of the zVps15 protein. Furthermore, the z*vps15* transcript was detected in zebrafish embryos from the eight-cell stage (1.25 h post fertilization (h.p.f.)) to the protruding-mouth stage (72 h.p.f.) (Supplementary Fig. 3A,B).

**Figure 3:**
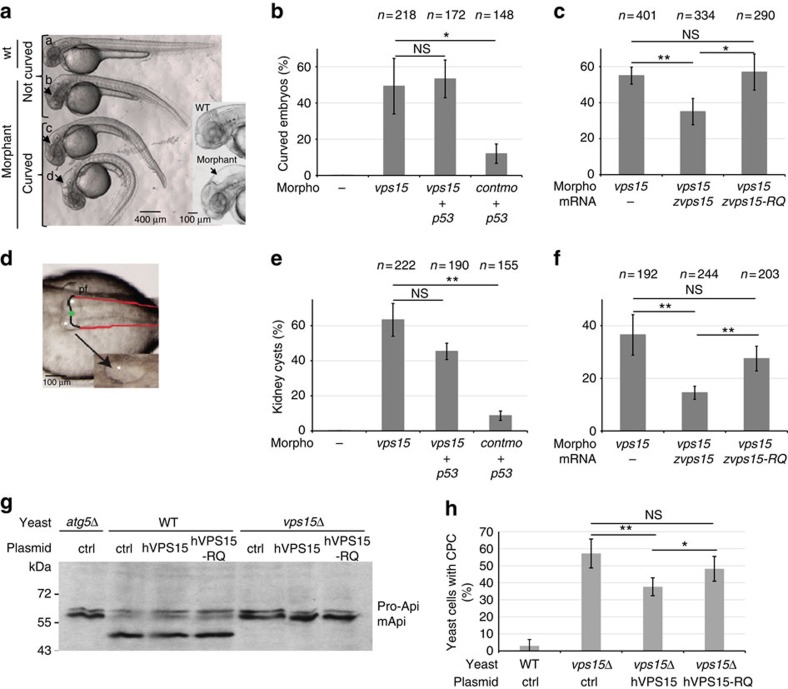
Zebrafish and yeast models to determine the VPS15-R998Q defects. (**a**) The *vps15* morphants injected with vps*15-mo* (b–d) show different degrees of body axis curvature and hydrocephaly (black arrows and zoom) compared with the control (*vps15cont*-mo; a). For quantification, only phenotypes c and d were considered curved. Fifty-six hours post-fertilization embryos were used. (**b**) Percentage of curved embryos among the population injected with different combinations of morpholinos (Morpho). (**c**) Percentage of curved embryos upon co-injection with vps*15-mo* plus zVPS15 wild-type (VPS15) or zVPS15-RQ mRNA. In **b** and **c**, *n* indicates the number of injected embryos that were counted. Statistical significance was determined using the Student's *t*-test, NS, non-significant, *P<0.05, **P<0.01 (**d**) Pronephric cysts (white stars) were observed in *vps15* morphants. Black line: pronephric tubule; green dot: glomerulus; red line: pronephric duct; pf: pectoral fin. (**e**) Percentage of embryos forming kidney cysts upon injection with different combinations of morpholinos. (**f**) *vps15* morphants co-injected with VPS15 wild-type or VPS15-RQ mRNA. In **e** and **f**, *n* indicates the number of injected embryos that were counted. Statistical significance was determined using the Student's *t*-test, NS, non-significant, **P<0.01 (**g**) The wild-type (WT), *atg5Δ* and *vps15Δ* yeast cells bearing ctrl, hVPS15 or hVPS15-RQ plasmid, were grown under nitrogen deprivation for 4 h to induce autophagy and then collected. Western blot was performed to show the immature (proApi) and mature (mApi) forms of the vacuolar protease Api (Aminopeptidase 1). (**h**) *vps15Δ* yeast cells were transformed with the empty (ctrl), the wild-type hVPS15 or the mutant hVPS15-R998Q plasmid and the percentage of cells displaying an additional CMAC-positive compartment (CPC) was determined. At least 100 cells per experiment and transformant were counted. Graph shows mean of three experiments. Statistical significance was determined using the Student's *t*-test, NS, non-significant, *P<0.05, **P<0.01. All the data shown in the figure are from at least three independent experiments and error bars represent s.d.

To reveal the spatial expression pattern of z*vps15*, we performed whole-mount RNA *in situ* hybridization at 48 h.p.f. Expression of z*vps15* was observed in the head as well as in the pronephric duct (Supplementary Fig. 3C). Morpholinos directed against the *zvps15* start codon (*vps15*-mo) and control morpholino (*vps15cont*-mo) with five-base mismatch were synthesized. The *vps15*-mo inhibited specifically protein synthesis of a zVps15–GFP fusion protein (Supplementary Fig. 3D). At 56 h.p.f., 50% of the *vps15* morphants presented a severe curvature of the body axis (Fig. 3a,b). Co-injection of *vps15*-mo or *vps15cont*-mo with a morpholino directed against p53 messenger RNA (mRNA; *p53*-mo) did not rescue the phenotype (Fig. 3b), showing that the phenotype was not due to morpholino off-targeting mediated through p53 activation^19^. Moreover, 90% of the *vps15* morphants showed hydrocephalus (Fig. 3a). We also observed the presence of kidney cysts as well as a cystic dilatation of the region slightly posterior to the ear at the level of the pectoral fin in 61% of *vps15* morphants (Fig. 3d,e and Supplementary Fig. 3E), a phenotype not due to activation of p53-mediated apoptosis (Fig. 3e). In most morphants, we could detect two bilateral pronephric cysts (Fig. 3d).

To assess the specificity of the morpholino effects and test the patient VPS15-R998Q missense mutation, we determined whether the *vps15*-mo phenotypes associated with cilia dysfunctions (body axis curvature and kidney cysts) could be rescued by co-injection of a synthetic z*vps15* or z*vps15-R976Q* mutant mRNA (Fig. 3c,f). Co-injection of *zvps15* wild-type mRNA caused a statistically significant rescue of both phenotypes (body axis curvature and kidney structure), while co-injection of *zvps15-R976Q* mutant mRNA did not ameliorate the phenotypes displayed by the *vps15* morphants (Fig. 3c,f). These results show that the *zvps15-R976Q* mutant does not complement the ciliopathy phenotypes induced by *zvps15* depletion.

### In yeast VPS15-R998Q is linked to trafficking defects

To better understand the origin of the ciliopathy linked to the hVPS15 mutation, we studied the heterologous expression of hVPS15 and hVPS15-R998Q mutant in yeast. Human and yeast VPS15 proteins share only 33% identity and the R998 residue is not conserved in yeast ScVps15 protein^15^. Although a specific amino acid is frequently not conserved in yeast, the structure and the function of yeast and human proteins as a whole are closely related. Humanization of yeast cells by replacing the yeast gene by its human counterpart is a potent approach used to gain insights into the cellular role of the human protein^20^,^21^. In humanized yeast cells, we analysed the VPS15-dependent cellular functions such as growth, intracellular trafficking from the Golgi to the vacuole (VPS pathway) and autophagy. Wild-type (WT) or *vps15***Δ** mutant yeast cells were transformed by plasmids bearing either the wild-type hVPS15 or mutant hVPS15-R998Q cDNA, allowing either expression (CEN plasmid) or overexpression (2 μ plasmid) of the human cDNA, as controlled by western-blot (Supplementary Fig. 3F). We observed growth delays upon overexpression of hVPS15 suggesting that hVPS15 might hijack important cellular functions (Supplementary Fig. 3H). Autophagy was investigated by monitoring the maturation of the aminopeptidase Ape1, a common read-out of yeast autophagy^22^. We observed that as previously described autophagy is blocked in the *vps15***Δ** yeast mutant cells compared with the wild-type cells, indeed the aminopeptidase (Api) is not matured (Fig. 3g). However, although autophagy was impaired in *vps15***Δ** cells (Fig. 3g), this defect was not rescued by expression of hVPS15 wild-type or R998Q mutant (Fig. 3g). Transport to the vacuole along the VPS pathway was then analysed by using the CMAC fluorescent probe that normally only labels the lumen of the yeast vacuole (Supplementary Fig.3G). However, *vps15***Δ** cells show an additional CMAC-positive compartment (CPC; white arrows, Supplementary Fig. 3G) that was not rescued by the expression of hVPS15-R998Q mutant as efficiently as by the wild-type hVPS15 (Fig. 3h), indicating a default in transport to the vacuole. These yeast data suggest that the R998Q mutation might impair an *in vivo* function of hVPS15 in intracellular trafficking from the Golgi to the vacuole.

### VPS15 and VPS15-R998Q bind to their partners and to GM130

**Figure 4:**
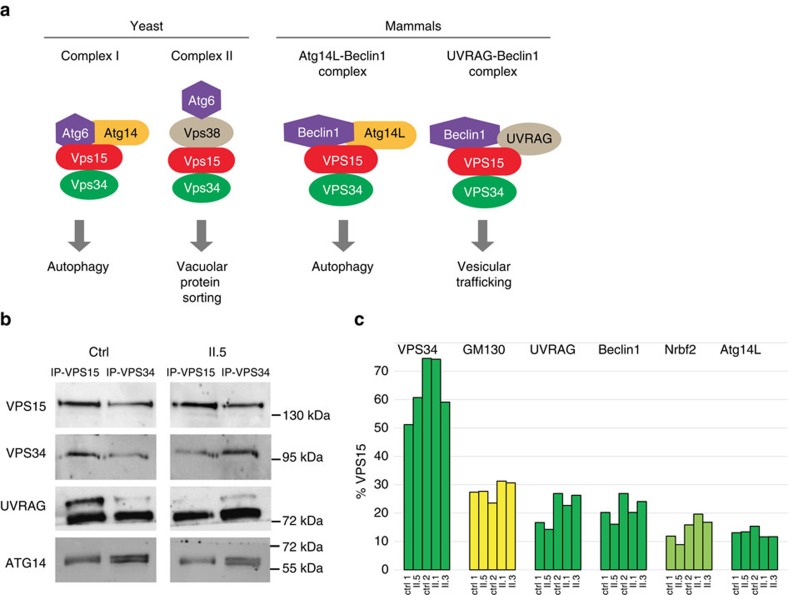
Identification of VPS15 protein complexes. (**a**) The VPS15 protein complexes are similar in their organization and function in yeast and mammals. (**b**) Immunoprecipitations with VPS15 or VPS34 antibodies were done on cell lysates from control (ctrl) or patient (II.5) fibroblasts and known VPS15 and VPS34 interaction partners (VPS34, VPS15, UVRAG and ATG14) were detected by western blot. (**c**) Mass spectrometry data of VPS15 interaction partners were analysed, using the number of spectra found for VPS15 as a reference (100%). The number of spectra found for VPS34, GM130, UVRAG, Beclin1, Nrbf2 and Atg14L in the different samples (ctrl 1 and 2 and patient II.1, II.3 and II.5) was expressed relatively to the number of spectra found for VPS15 and percentages plotted on a histogram.

Having shown that VPS15-R998Q leads to ciliary phenotypes, we next investigated whether observed defects were due to a loss of protein interaction. Indeed, VPS15 is known to be involved in two distinct cellular functions when interacting with different proteins, the UVRAG/Beclin1 complex involved in endosomal trafficking and the Atg14L/Beclin1 complex required for autophagy (Fig. 4a)^10^,^11^. Endogenous VPS15 or VPS15-R998Q was immunoprecipitated from either control or patient fibroblasts in rich (+FCS) or serum-deprivation conditions (−FCS) to induce cilium formation and co-immunoprecipitation of VPS34 and Beclin1 proteins assessed by western blot (Supplementary Fig. 4A). The VPS15 protein was detected in the input of control and patient cells (in rich and starved conditions) indicating that the R998Q substitution does not hamper the stability of VPS15-R998Q. Moreover, VPS34 and Beclin1 proteins interacted with VPS15 and VPS15-R998Q proteins in both growth conditions (+ and −FCS), suggesting that the VPS15–VPS34 complex is functional and active to produce PtdIns3P. Indeed, PtdIns3P-positive structures were detected in the control and patient fibroblasts transfected with the 2XFYVE-GFP probe (Supplementary Fig. 4B) that is specific for PtdIns3P (ref. 23).

**Figure 5:**
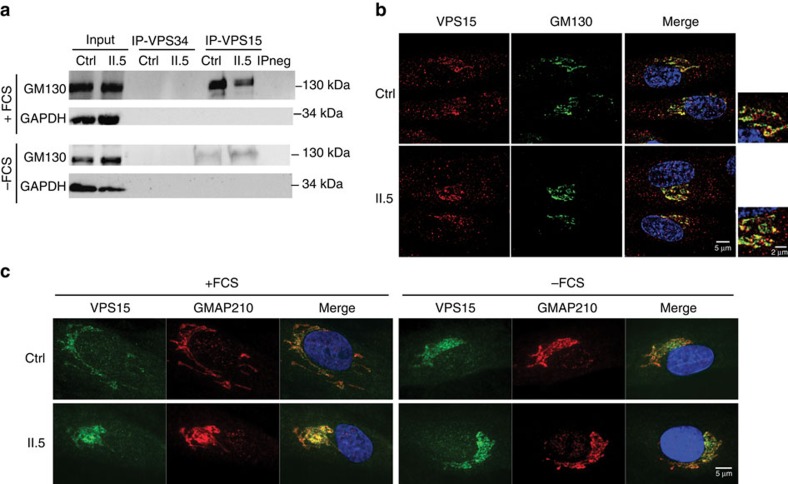
GM130 interacts with VPS15 but not VPS34. (**a**) Control and patient fibroblasts were grown in complete medium (+FCS) or deprived of serum (−FCS) for 24 h. Immunoprecipitations with VPS15 or VPS34 antibodies were done on cell lysates and GM130 was detected by western blot. GAPDH was used as a loading control. (**b**) Control (ctrl) and patient (II.5) fibroblasts were grown and deprived of serum for 24 h. Immunofluorescence against VPS15 (red) and GM130 (green) and DAPI staining (blue) was performed and the cells were observed with a confocal microscope. (**c**) Control (ctrl) and patient (II.5) fibroblasts were grown in complete medium (+FCS) or deprived of serum (−FCS) for 24 h. Immunofluorescence against VPS15 (green) and GMAP210 (red) and DAPI staining (blue) was performed and the cells were observed with a confocal microscope.

To further analyse the VPS15 protein complexes, VPS15 immunoprecipitation was done on control and patient cells in rich conditions (+FCS), proteins specifically retained on the VPS15-magnetic beads and not on naked beads (IPneg) were determined by mass spectrometry (Fig. 4b and Supplementary Fig. 4C). As previously shown, high amounts of VPS34 and UVRAG were found interacting with VPS15 (control) and VPS15-R998Q (patients II.1, II.3 and II.5), and so were Beclin1 and Atg14L, two known interactors of VPS15 (Fig. 4b and Supplementary Fig. 4C). We also found NRBF2 (Supplementary Fig.4C), a protein recently identified as belonging to the Atg14L–Beclin1 complex^24^. Protein abundance estimated from the number of mass spectrometry spectra (Supplementary Fig. 4C) shows that between the two controls and the three patients, the efficiency with which the different proteins (VPS34, UVRAG, Beclin1, NRBF2 and Atg14L) were immunoprecipitated with VPS15 are similar (Fig. 4c). This suggests that the R998Q substitution does not destabilize specifically one of these protein interactions. Interestingly, the *cis*-Golgi protein GM130 (GOLGA2) was also found among the strongest interactors of wild-type and mutant VPS15 (Fig. 4c and Supplementary Fig. 4C). GM130 has not been previously reported as interacting with VPS15 ( http://thebiogrid.org). However, this new interaction is highly relevant because the *cis*-Golgi is involved in transport to the cilium and GM130 is required for Golgi morphology and ciliogenesis via its interaction with the centrosomal protein AKAP450 (ref. 25). To confirm our mass spectrometry data, we tested whether GM130 can be detected by western blot after immunoprecipitation of VPS15 from control and patient (II.5) cells grown in complete medium (+FCS) or deprived of serum (−FCS; Fig. 5a). We also immunoprecipitated VPS34 from the same protein extracts to determine whether VPS34 was interacting with GM130 (Fig. 5a). As controls, VPS15, VPS34, UVRAG and Atg14L were detected by western blot in the same samples (+FCS; Fig. 4b). The results show that only VPS15 is specifically interacting with GM130, whereas in the same conditions VPS34 does not interact with GM130 but retains binding to VPS15, UVRAG and Atg14L (Figs 4b and 5b). To further confirm this result, we did the reverse immunoprecipitation experiment and detected VPS15 by western blot in control and patient fibroblasts after GM130 immunoprecipitation (Supplementary Fig. 5C).

To determine the intracellular localization of VPS15 in ciliary conditions, control, VPS15-R998Q patient II.5 and *BBS4*^*−/−*^ patient fibroblasts were grown in medium without FCS, fixed and labelled for VPS15 (red) and acetylated α-tubulin (green) before observation (Supplementary Fig. 5A). The primary cilium is present after serum deprivation and there is no co-localization between VPS15 and acetylated α-tubulin in patient and control cells (Supplementary Fig. 5A). Indeed, VPS15 is rather concentrated around the nucleus, suggesting Golgi localization. The intracellular localization of VPS15 was also analysed by staining VPS15 (red) and GM130 (green; Fig. 5b). In confocal microscopy, this staining shows that the pool of VPS15 present at the Golgi co-localizes with the golgin GM130 in control and patient cells. Since IFT20 required for ciliary assembly is anchored at the *cis*-Golgi by the golgin GMAP210 (ref. 5), immunofluorescence staining against VPS15 and GMAP210 was done on control (ctrl) and patient II.5 fibroblasts grown in complete medium (+FCS) and after serum deprivation (−FCS; Fig. 5c). This staining confirms the localization of VPS15 at the Golgi in both rich and ciliary conditions. In contrast, and as already suggested by the interaction results (Fig. 4b,c), VPS34 did not co-localize with GM130 at the Golgi (Supplementary Fig. 5B). These results indicate that the PtdIns3P kinase VPS34 does not belong to the GM130–VPS15 *cis*-Golgi localized protein complex. We decided to further investigate the cilium-related role of VPS15 at the Golgi.

### IFT20 release from the Golgi is reduced in VPS15-R998Q cells

**Figure 6:**
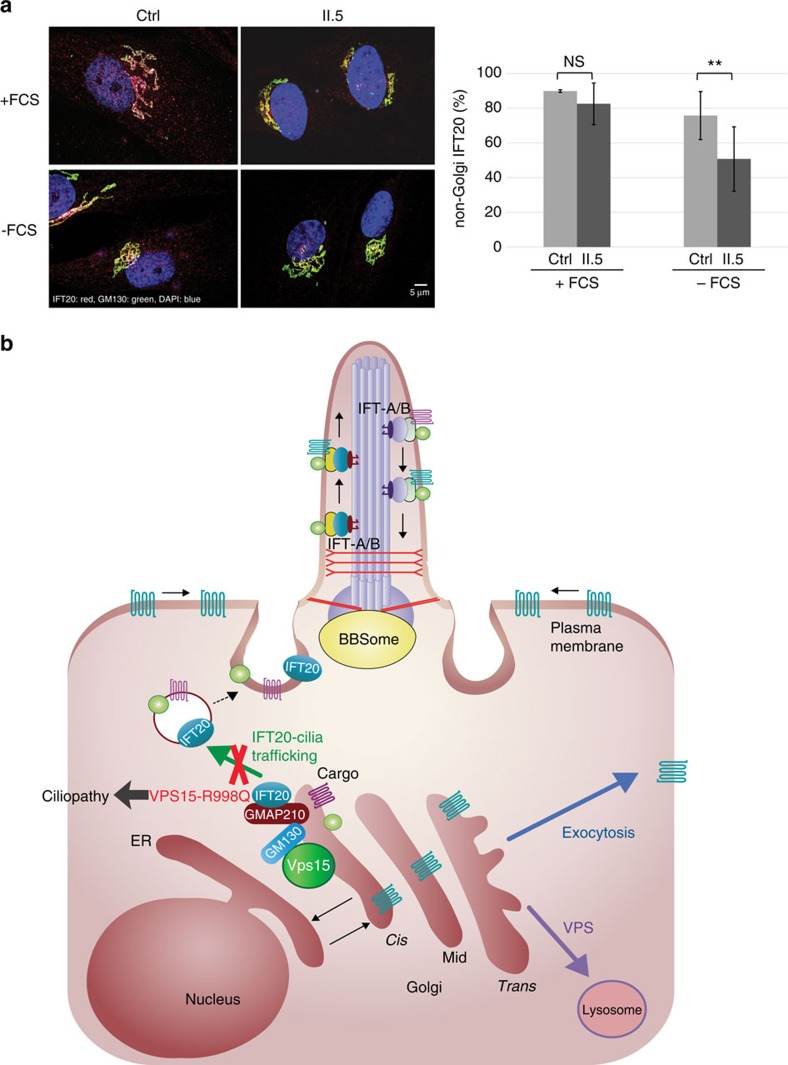
IFT20 is retained in the Golgi in patient fibroblasts. (**a**) Control (ctrl) and patient (II.5) fibroblasts were grown in complete medium (+FCS) or deprived of serum for 24 h (−FCS) and fixed. Immunofluorescence against IFT20 (red) and GM130 (green) and DAPI staining (blue) was performed and the cells were observed on a confocal microscope, only the merge (IFT20, GM130 and DAPI) is shown. Amount of red (IFT20) fluorescence in the cytoplasm was determined using ImageJ to measure the total amount of IFT20 fluorescence and substract fluorescence at the Golgi (green, GM130). The measures were done on 10 non-serum-deprived cells and 26 and 28 cells for ctrl and patient cells in serum-deprived conditions, respectively and error bars represent s.d. Mean fluorescence was calculated and statistical significance determined using a Student's *t*-test, **P<0.001. (**b**) Schematic representation of intracellular trafficking pathways to deliver cargo proteins to the ciliary base. The role of the VPS15 protein at the *cis*-Golgi is based on our data.

Some ciliary cargoes are transported from the *cis*-Golgi to the base of the cilia in IFT20-dependent vesicles. The IFT20 protein, belonging to the IFT-B complex, is anchored at the *cis*-Golgi by the golgin GMAP210 (refs 5, 6), which is redundant with GM130 for *cis*-Golgi cargo delivery^26^. Moreover, partial depletion of IFT20 induces a shorter length of the primary cilia^27^, a phenotype similar to the one observed in VPS15-R998Q cells (Fig. 2). Therefore, we investigated the localization of IFT20 by immunofluorescence in control and patient fibroblasts upon cilium induction (−FCS) or not (+FCS). In cells grown in complete medium (+FCS), IFT20 (red) was detected in vesicles distributed throughout the cytoplasm and at the *cis*-Golgi where it co-localized with GM130 (green). In these conditions, IFT20-positive vesicles (red) were observed in patient and control cells (Fig. 6a and Supplementary Fig. 6). However, upon serum deprivation, while the distribution of IFT20 was similar to the complete medium conditions in control cells (vesicles and *cis*-Golgi), significantly less non-Golgi (vesicular) IFT20 was observed in the patient fibroblasts (Fig. 6a). Indeed, in the latter, IFT20 localization seems restricted to the Golgi (Fig. 6a and Supplementary Fig. 6A,B). We confirmed this Golgi localization in patient cells by performing an immunoprecipitation of IFT20 followed by a western blot against GMAP210. In both, control and patient fibroblasts, the interaction between IFT20 and its anchor GMAP210 (ref. 5) was observed (Supplementary Fig. 6C), confirming that the R998Q missense mutation does not disrupt the IFT20–GMAP210 interaction. Thus, the defect observed in patient fibroblasts seems to be localized at the level of the formation and/or release of IFT20 positive vesicles from the *cis*-Golgi. The difference between control and patient fibroblast is small but significative, and even a slight decrease in transport of cargo to the cilium may result in a decreased cilium growth and/or altered signalling.

Overall, our results support a new role for VPS15, independently of VPS34, in the formation/development of the primary cilium, presumably by altering the IFT20-dependent Golgi to cilium vesicular transport as this pathway is affected in cells from patients bearing the R998Q mutation in *VPS15*.

## Discussion

Here, we report a missense mutation in the *VPS15* gene identified in a unique family presenting with early-onset retinal degeneration, late childhood kidney failure associated to mild skeletal developmental features with moderate intellectual disability. Overall, this clinical presentation, compatible with a ciliopathy, showed some overlap mainly with the well-known Senior–Loken and Bardet–Biedl syndromes^2^. More than a 1000 proteins are probably required for the biogenesis and function of the vertebrate primary cilium and more than a 100 genes are now identified as mutated in ciliopathies ( http://www.omim.org). Over the past 10 years, classical gene identification optimized now by next-generation sequencing strategies have allowed the identification of ciliary genes carrying mutations within different families such as, for example, BBS syndrome (mutations in *BBS1* or *BBS10* accounting each for 20% of the families)^28^. Nowadays, new ciliopathy causative genes are very likely to be identified in very few if not unique families. One explanation could be that the gene involved may code for an essential protein for which only a very limited number of mutation sites may be tolerated, the remaining mutational sites being probably lethal. Here, we report the first family with a missense mutation in the *VPS15/PIK3R4* gene. In metazoan (mouse and Drosophila), *VPS15* is an essential gene^11^,^12^. The R998Q substitution is predicted to be probably damaging by the PolyPhen-2 programme^13^. Indeed, arginine is a positively charged polar amino acid, frequently involved in salt-bridges and important for protein binding sites. Thus, a change from an arginine to a glutamine that is polar and uncharged is certainly not neutral. The recently solved crystal structure of the yeast Vps15–Vps34 complex required for trafficking shows that the Vps15 kinase domain interacts with the Vps34 kinase domain to regulate its activity^15^. The WD40 repeat domain of Vps15 is engaged in interactions that bridge the Vp15/Vps34 heterodimer with the Atg6/Vps38 sub-complex^15^. On the basis of this structure, the kinase domain of Vps15 is probably inactive once bound to Vps34 because its ATP binding site is not accessible^15^, however in the human VPS15–GM130 complex this kinase domain might be functional.

The link between VPS15 and cilia was not obvious since VPS15 was mostly shown to be involved in membrane trafficking and autophagy (Fig. 4). However, it has long been hypothesized that VPS15 may have additional roles, because yeast results indicate that some VPS15 acts independently of the two UVRAG–Beclin1 and ATG14L–Beclin1 complexes^22^. Now with the clear ciliopathy phenotypes from human patients and zebrafish knockdown shown in this work, an additional function in ciliogenesis or cilia function is suggested. Functional assays in patient cells show that the VPS15-R998Q mutation affects primary cilia length and IFT20-dependent trafficking from the *cis*-Golgi. The retinal degeneration could be due to a trafficking defect at the level of photoreceptor cells that are ciliated sensory cells. Indeed, phototransduction proteins such as rhodopsin are transported through the connecting cilium structure to the outer segment by vesicular trafficking involving the IFT machinery^29^. IFT20 is required for assembly and maintenance of the photoreceptor outer segment and likely participates both as component of IFT particle and as a *cis*-Golgi specific effector independent of canonical IFT machinery^30^. On the basis of retinal phenotypes in patients, we hypothesize that VPS15 is a novel actor in the photoreceptor transport machinery. Further investigations are necessary to define the role of VPS15 compared with other effectors involved in this trafficking. VPS15 could also be a novel regulator of the ciliary transport machinery in the kidney tubules, since a conditional knockout for IFT20 in mice shows rapid development of kidney cysts^31^.

Here, we have identified a novel protein complex localized at the *cis*-Golgi and encompassing VPS15 and the golgin GM130. This new VPS15–GM130 complex could be involved in IFT20-dependent trafficking to the cilium (Fig. 6b). Indeed, a pool of IFT20 is associated with the *cis*-Golgi where it co-localizes partially with GM130 with increased IFT20 release from the Golgi in control fibroblast than in patient cells. Golgi-associated IFT20 is required for cilia assembly^6^. Interestingly, in mammalian cells, strong IFT20 knockdown results in a lack of cilia assembly^6^ and weaker IFT20 knockdown in shorter primary cilia^27^ and impaired transport of cargo to the cilia^6^. Thus, even a moderate decrease in the amount of IFT20 involved in the *cis*-Golgi to cilium transport could have a tremendous effect on the primary cilium assembly and function.

Golgins are key Golgi effectors since they participate to the specificity of intracellular trafficking by capturing vesicles of different origins^32^. At the *cis*-Golgi level, the golgin GMAP210 serves as an anchor for IFT20 (ref. 5) and knockdown of GMAP210 leads to a defect in cilia formation^5^. However, the knockdown of GM130 has been described as not affecting cilia assembly^33^. Interestingly, recent data show that GMAP210 is redundant with GM130 for cargo delivery to the *cis*-Golgi^26^. Indeed, among the 10 widely conserved golgins tested, only GM130 and GMAP210 were shown to be specific for tethering vesicles arriving at the *cis*-Golgi from the endoplasmic reticulum^32^. Thus, more redundancy in the function of these two proteins might exist, especially at the level of primary cilium formation/function. Another argument playing in favour of a role of GM130 in ciliogenesis is its interaction with the centrosomal protein AKAP450, an interaction required for ciliogenesis and Golgi integrity^25^. Thus, GM130 role may be less direct than GMAP210 and could involve additional proteins. Here, we show that the VPS15 kinase interacts with GM130 and that in VPS15-R998Q patient cells IFT20 is partially retained in the Golgi. Thus, the VPS15–GM130 protein complex could serve as platform to form IFT20-positive vesicles targeted from the *cis*-Golgi to the cilium. Mass spectrometry analyses aimed at unravelling the molecular mechanism of VPS15 function in cilium formation indicate that indeed, although the VPS15–GM130 interaction is conserved between control and patient fibroblasts, a GM130–IFT20 interaction was detected in control fibroblasts but never in patient cells (by three independent experiments). Additional research along this line of work is needed to confirm this hypothesis.

Misbalanced trafficking to the cilia has dramatic consequences on cilia assembly and function, resulting in ciliopathies. Among the trafficking effectors involved, the BBSome, a complex formed by a subset of BBS proteins, is altered in a significant number of patients with Bardet–Biedl syndrome. The cilia trafficking pathway is still poorly understood, yet very important for proper signalling functions^4^. During evolution and to ensure intracellular trafficking, different coat complexes have assembled; among them are the clathrin, COPI/COPII and BBSome coat that share a common overall structure^8^. The VPS15–VPS34 platform is crucial for trafficking and this assembly platform is well conserved during evolution. VPS15 could also be involved at the *cis*-Golgi to serve as a platform for ciliary-targeted membrane proteins. Indeed, GMAP210 and GM130 golgins are not specific for ciliary cargoes, thus other proteins are involved to control this specificity and VPS15 or an interaction partner could be good candidates (Fig. 6b).

Overall, our results support a new role for VPS15, independent of VPS34, in the IFT20-dependent Golgi to cilium vesicular transport as this pathway is affected in patients bearing the VPS15-R998Q mutation. However, VPS15 does not directly interact with IFT20 and its interaction with GM130 is not hampered by the presence of the point mutation either. Therefore, subsequent work will be needed to analyse the interactions between IFT20 and the VP15–GM130 complex to confirm VPS15-dependent IFT20–GM130 interaction. Thus, the potential involvement of partners of the VPS15–GM130 complex whose function might be hampered by the patient mutation in VPS15 will need further investigations.

## Methods

### Ethical approval

After informed consent of the patient and his/her representative according to the French legislation, peripheral blood samples were obtained from the affected children and their parents as well as control individuals. DNA from all the collected samples was extracted according to standard procedures. The objectives and the aim of the study were clearly explained to the patients and this study was approved by the local ethics committee at Hôpitaux Universitaires de Strasbourg (Strasbourg University Hospital).

### Cell cultures

Fibroblasts of patients and age-matched healthy control individuals were obtained by skin biopsy, as previously described^34^. Primary skin fibroblasts from VPS15-R998Q patients and age-matched healthy control skin fibroblasts, as well as the hTERT-RPE1 cell line were grown in DMEM supplemented with 10% fetal calf serum (FCS) and 1% PSG (penicillin–streptomycin–glutamin). To induce primary cilium formation, the cells were deprived of serum by growth for 24 h in DMEM with 1% PSG but only 0.1% FCS (conditions: −FCS).

### 250 K Affymetrix array and Sanger sequencing

To identify the genetic mutation responsible for the disease, we screened a panel of 30 ciliopathy-related genes by targeted exon-capture strategy coupled with multiplexing and high-throughput sequencing^35^, and we found no mutations. Then, we performed a genome-wide homozygosity mapping using GeneChip Human 250 K SNP Affymetrix (Supplementary Fig. 1) combined to whole-exome sequencing (Agilent SureSelect All Exon XT2 50 Mb kit) of the three affected siblings (II.1, II.3, II.5) and the healthy sister (II.4), using the facilities provided by IntegraGen (Evry, France). Sequencing data processing and variant calling (SNP and InDels) with the Genome Analysis Toolkit revealed 60,612 to 63,887 genetic variants per proband (Supplementary Table 1). Variant filtering was performed with the VaRank programme^36^ using stringent criteria excluding (i) non-pathogenic variants defined in dbSNP 138, (ii) variants represented with an allele frequency of more than 1% in dbSNP, the Exome Variant Server, the Thousand Genomes Project Catalog and the ExAC database, (iii) variants found in the homozygous state or more than once in the heterozygous state in 70 control exomes, (iv) variants into 5′-UTR (untranslated region), 3′-UTR, downstream, upstream or intron locations without local splice effect prediction, (v) synonymous variants without local splice effect prediction. This step reduced the number of genetics variants to 1,085, 793, 1,124 and 2,009 variants, respectively, per proband (Supplementary Table 1). The variant and its cosegregation with the phenotype in the family was confirmed by Sanger sequencing. Sanger sequencing was performed by way of PCR amplification with 50 ng of genomic DNA template. The primers were designed with Primer 3 ( http://frodo.wi.mit.edu/primer3) and are detailed in Supplementary Table 2. Bidirectional sequencing of the purified PCR products was performed by GATC Biotech. Segregation analysis ruled out the mutations in the RECQL4/MFSD3 gene as they were both carried in *cis* by a maternal chromosome. The very closely related intronic AK9 insertions of six or four nucleotides (NM_001145128.2:c.5315+104_5315+105insAGAGAG and NM_001145128.2:c.5315+106_5315+107insAGAG) in a repetition of an AG motif in intron 38 are present in the three patients and in the healthy sister and thus not specific to the disease.

### Zebrafish experiments

Fish were bred and raised at 28.5 °C as described previously^37^. The AB wild-type line (University of Oregon, Eugene) was used for all the experiments. Sexually mature fish were crossed in couples, and the eggs were collected after being laid. For experiments, fertilized eggs were raised in 1 × Instant Ocean salt solution (Aquarium Systems, Inc.) supplemented with 200 μM 1-phenyl 2-thiourea to suppress melanogenesis.

The *pik3R4/vps15* full-length sequence was amplified with the pikwt-forw and pikwt-rev primers (Supplementary Table 2) and cloned into pCS2+GFP with EcoRI-XhoI. pGEM T-easy vector containing the 1,100 bp fragment used for whole mount was cut with EcoRI, and the resulting fragment cloned into pCS2+GFP to give the zVps15-GFP plasmid. For *zvps15-RQ* mutant, we amplified the wild-type sequence with pikmut-forw/pikwt-forw and pikmut-rev/pikwt-rev (Supplementary Table 2). The two obtained fragments were then amplified together with a mix of pikwtforw/pikwtrev and finally cloned into pCS2+GFP with EcoRI-XhoI.

RT–PCR (PCR with reverse transcription) was carried out following standard protocol. Total RNA was isolated from 24 to 72 h.p.f. embryos using Tri-reagent (Invitrogen, Carlsbad, CA, USA). For *zvps15 in situ* hybridization, we used as probe a fragment of 1,100 bp amplified by PCR with the following primers pik3r4WM-forw and pik3r4WM-rev and cloned into pGEM-T-easy vector (Promega). For RNA probe, we used NcoI and SP6 RNA polymerase. RT–PCR was done with the same primer pairs.

Whole-mount *in situ* hybridization was performed as previously described^38^. To prevent pigmentation for expression analysis after 24 h.p.f., embryos were transferred to water containing 0.2 mM 1-phenyl-2-thiourea at 20 h.p.f. and fixed at appropriate stages.

For injections, the zebrafish eggs were collected shortly after being laid. The cleaned eggs were transferred to a petri dish with a minimal amount of water. The embryos were injected (FemtoJet; Eppendorf) through the chorion into the yolk at the one-cell stage with 12 nl of solution. The injection needles were pulled from borosilicate glass capillary tubes with filament (Warner Instruments) using a micropipette puller (Sutter Instrument Co). Morpholinos (Gene Tools, LLC) were injected at the following concentrations: *vps15*-mo: 5′-AGTTGGTTCCCCATCTCACTGGATC-3′ (0.4 mM); p53-mo: 5′-GCGCCATTGCTTTGCAAGA-ATTG-3′ (0.4 mM); *vps15cont*-mo: 5′-AGTaGcTT CCCgATCTCAgTcGATC-3′ (0.4 mM). All the dilutions were made in distilled water. Phenol red was added to the samples before injection (0.1% final concentration). zVps15-GFP plasmid was injected at the final concentration of 40 ng μl^−1^. *zvps15* wild-type and *zvps15-R976Q* mRNA were injected at the final concentration of 40 ng μl^−1^.

### Materials and methods for yeast cells

The R998Q mutation was introduced into the hVPS15 cDNA by polymerase chain reaction (PCR) with Phusion High-Fidelity DNA polymerase (Thermo Scientific) on the pDONR223 entry vector bearing hVPS15 cDNA (Addgene 23488). The resulting pDONR223-hVPS15-R998Q or pDONR223-hVPS15 were cloned by the Gateway LR reaction (Invitrogen) into yeast destination vectors (Addgene plasmid numbers 14,196 and 14,252; ref. 39) to obtain the pSF194 (pAG423-promGPD-hVPS15-R998Q) and pSF196 (pAG413-promGPD-hVPS15-R998Q) or pSF198 (pAG423-promGPD-hVPS15) and pSF199 (pAG413-promGPD-hVPS15) plasmids. All the plasmids sequences were verified by sequencing (GATC Biotech).

*S. cerevisiae* strains used in this study are BY4742 WT (*MATα leu2*Δ*0 ura3*Δ*0 his3*Δ*0 lys2*Δ*0*), *vps15Δ* (BY4742 *vps15*::*kanMX*) and *atg5Δ* (BY4742 *atg5*::*kanMX*). The indicated yeast strain were grown at 30 °C to mid-exponential growth phase in rich medium (YPD): 1% yeast extract, 2% peptone, 2% glucose or in synthetic-defined medium: 0.67% yeast nitrogen base (YNB) without amino acids, 2% glucose and the appropriate dropout mix. Autophagy was induced by incubation for 4 h in synthetic-defined-N medium: 0.17% (YNB) without ammonium sulfate, 2% glucose. Yeast cells were transformed using the modified lithium acetate method.

Living cells expressing hVPS15 or hVPS15-R998Q were harvested at an *D*_600nm_ 0.5–1 and resuspended in synthetic complete yeast medium before visualization. For CMAC (Invitrogen) staining, the indicated yeast strain was harvested by a 500*g* centrifugation for 1 min, resuspended in synthetic defined medium and stained with CMAC (33 μM) for 10 min at 30 °C before washing with phosphate-buffered saline (PBS). Observation was performed with 100X/1.45 oil objective (Zeiss) on a fluorescence Axio Observer D1 microscope (Zeiss) using DAPI filter and DIC optics. Images were captured with a CoolSnap HQ2 photometrix camera (Roper Scientific) and treated by ImageJ (Rasband W.S., ImageJ, U.S. National Institutes of Health, Bethesda, MD, USA, http://imagej.nih.gov/ij/). For western-blot analysis, total yeast extracts were obtained by NaOH lysis followed by TCA precipitation as previously described^40^. The equivalent of 1.5 *D*_600nm_ unit of yeast cells were resuspended in 50 μl of 2X Laemmli buffer plus Tris Base. The samples were incubated for 5 min at 37 °C and analysed by 10% SDS–polyacrylamide gel electrophoresis followed by immunoblotting with anti-hVPS15 (Novus Bio, NBP1-30463) or anti-Ape1 (also termed Api, kind gift from Daniel Klionsky) using the standard procedures. The images were acquired with the ChemiDoc Touch Imaging System (Bio-Rad).

### Immunofluorescence

Primary fibroblasts from patients and control individuals were grown in Nunc Lab-Tek chamber slides (Thermo Scientific) or on spot slides, deprived of FCS for 24 h (or not) and fixed with 4% paraformaldehyde. After incubation with 0.5% Triton X-100 for 10 min, and blocking in PBS-20% FCS, the cells were incubated for 1 h with primary antibodies, washed three times in PBS, incubated for 1 h with secondary antibodies and DAPI, washed again in PBS and mounted in Elvanol No-Fade mounting medium before observation. Primary cilia were labelled with an antibody directed against acetylated α-tubulin highlighting the axoneme^41^. Primary antibodies against acetylated α-tubulin (Abcam, ab24610), VPS15 (Novus Bio, NBP1-30463), VPS34 (Cell Signaling, 4263), GM130 (Abcam, ab169276), GMAP210 (Thermo Scientific, MA1-23294), IFT20 (Proteintech, 13615-1-AP) and HA (Roche, 1867423) were used. Secondary antibodies were goat anti-mouse Alexa Fluor coupled (either 488 or 568) IgG (Invitrogen), goat anti-rat Alexa Fluor 488 coupled IgG (Abcam, ab150157), donkey anti-rabbit IgG-FITC (Santa Cruz sc-2090) and donkey anti-mouse IgG (H+L) FITC conjugate (Thermo Scientific A16012). The cells were observed on a fluorescence (Zeiss Axio Observer D1) or confocal (Zeiss LSM700, × 40 objective, Plateforme Microscopie et Imagerie (IBMP, Strasbourg, France)) microscope and images processed with ImageJ. The cilia length from the base to the tip was measured using the ImageJ program on confocal images, and the length of each primary cilia was determined for 120 to 200 cells per sample (ctrl1, ctrl2, ctrl3, II.1, II.3, II.5 and BBS4^−/−^).

### Transient transfection for GFP-2XFYVE^HRS^ and VPS15-HA plasmids

The fibroblasts cells were cultured to be 60% confluent the day of transfection in Nunc Lab-Tek chamber slides (Thermo Scientific). Five hundred nanograms of plasmid was transfected using the DNA transfection reagent (jetPEI, Polyplus transfection) according to the protocol for adherent cells. After 24 h, the fibroblast cells were washed three times in PBS, then the cells were fixed for 30 min at room temperature with 4% paraformaldehyde, rinsed three times in PBS for 5 min each, stained with DAPI and confocal fluorescence microscopy was done. Confocal microscopy was performed on a Zeiss LSM700 microscope. The GFP-2XFYVE^**HRS**^ plasmid was a kind gift from Harald Stenmark. The VPS15-HA plasmid was obtained by cloning the pDONR223-hVPS15 described above into the human destination vector pCSf107mT-GATEWAY-3′-3HA (Addgene, 67616) by the Gateway LR reaction (Invitrogen). Plasmid was verified by sequencing.

### Co-immunoprecipitation and mass-spectrometry analysis

The cells were grown in DMEM (Catalogue no. 31885; Gibco Invitrogen), 10% FBS and penicillin and streptomycin (P/S) to full confluence. To induce primary cilia, the cells were grown to confluence in DMEM containing 10% FCS, then washed with PBS and starved by serum deprivation for 24 h with DMEM 0.1% FCS. Before lysis, the cells were rinsed with PBS at 4 °C, resuspended in non-denaturing lysis buffer (20 mM Tris HCL pH 8; 137 mM NaCl; 1% Nonidet P-40; 2 mM EDTA) with a protease inhibitor cocktail (Roche 06538282001) and incubated on ice for 15 min under gentle shaking. The samples were then centrifuged at 12,000*g* for 20 min at 4 °C, protein concentration was measured using Qubit Protein Assay Kit (Life Technologies). Five hundred micrograms of cell lysates were incubated with VPS15 antibodies (Novus Biologicals NBP1-30463) on a rocker shaker overnight at 4 °C. The immunocomplexes were captured by protein G sepharose beads (Dutcher 17-0618-05) for 2 h at 4 °C under gentle shaking. Sepharose G beads were washed 6 × 5 min with non-denaturing lysis buffer with a protease inhibitor cocktail, resuspended in 2X Laemmli buffer and boiled for 10 min at 95 °C to dissociate the immunocomplexes from the beads. For western blot, the same primary antibodies as for immunofluorescence were used, and anti-GMAP210 (Thermo Scientific, #MA1-23294), anti-Beclin1 (Cell Signaling, #3495), anti-Atg14L (Cell Signaling, #5504), anti-UVRAG (Abcam, #ab174550) and anti-GAPDH (Abcam, #ab181602) antibodies were also used. For the VPS15-HA control experiment, immunoprecipitation was done with the anti-HA antibody used for immunofluorescence and western blot detection with a mouse anti-HA antibody (Abcam,ab130275). Uncropped images of the western blots are shown in Supplementary Fig. 7 and Supplementary Fig. 8.

For mass-spectrometry analyses, endogenous VPS15 immunoprecipitation was carried out with μMACS Protein A/G microbeads (Miltenyi Biotec) and VPS15 antibody (Novus Biologicals NBP1-30463), according to the manufacturer's protocol. Each protein sample was split in half. The second halves were used as negative controls, omitting antibodies during the immunoprecipitations (IPneg). Proteins complexes were eluted out of the magnetic stand with the SDS gel-loading buffer from the kit. Co-immunoprecipitation experiments were carried out in replicates for all the samples (two healthy controls and the three patients). The samples were prepared for mass spectrometry analyses as previously described^42^. Briefly, eluted proteins were precipitated with 0.1 M ammonium acetate in 100% methanol. After a reduction-alkylation step (dithiothreitol 5 mM–iodoacetamide 10 mM), proteins were digested overnight with 1/25 (W/W) of modified sequencing-grade trypsin (Promega, Madison, WI, USA) in 50 mM ammonium bicarbonate. Resulting peptides were vacuum-dried in a SpeedVac concentrator and re-suspended in water containing 0.1% FA (solvent A) before being injected on nanoLC-MS/MS (NanoLC-2DPlus system with nanoFlex ChiP module; Eksigent, ABSciex, Concord, Ontario, Canada, coupled to a TripleTOF 5600 mass spectrometer (ABSciex)). Peptides were eluted from the C-18 analytical column (75 μm ID × 15 cm ChromXP; Eksigent) with a 5–40% gradient of acetonitrile (solvent B) for 90 min.

The data were searched against the complete Human proteome set from the SwissProt database (released 2013/01/09; 43.964 sequences). Peptides were identified with Mascot algorithm (version 2.2, Matrix Science, London, UK) through the ProteinScape 3.1 package (Bruker). They were validated with a minimum score of 30, a *P* value <0.05 and a decoy database strategy was used to validate Mascot identifications at FDR<1%. For this study, selected protein partners were considered according to the following rule: presence in the five co-immunoprecipitation , absence from all the negative controls. They are sorted by decreasing average number of spectra.

### Antibodies

Detailed information on antibodies used in this study as well as their dilution are indicated in Supplementary Table 3.

### Data availability

The whole-exome sequencing data have been deposited at the European Genome-Phenome Archive (EGA), which is hosted by the European Bioinformatics Institute (EBI) and the Centre for Genomic Regulation (CRG), under accession number EGAS00001002075. The rest of the data that support the findings of this study are available from the corresponding authors on request.

## Additional information

**How to cite this article:** Stoetzel, C. *et al*. A mutation in *VPS15* (*PIK3R4)* causes a ciliopathy and affects IFT20 release from the *cis*-Golgi. *Nat. Commun.* **7,** 13586 doi: 10.1038/ncomms13586 (2016).

**Publisher's note**: Springer Nature remains neutral with regard to jurisdictional claims in published maps and institutional affiliations.

## Supplementary Material

Supplementary Information Supplementary Figures 1-8 and Supplementary Tables 1-3.
