# Supplementary material for: A mutation in VPS15 (PIK3R4) causes a ciliopathy and affects IFT20 release from the cis-Golgi
Source: Nat Commun. 2016 Nov 24;7:13586. doi: 10.1038/ncomms13586 (PMC5123056; doi:10.1038/ncomms13586)
Supplement: Supplementary Information — Supplementary Figures 1-8 and Supplementary Tables 1-3. [file ncomms13586-s1.pdf]

A

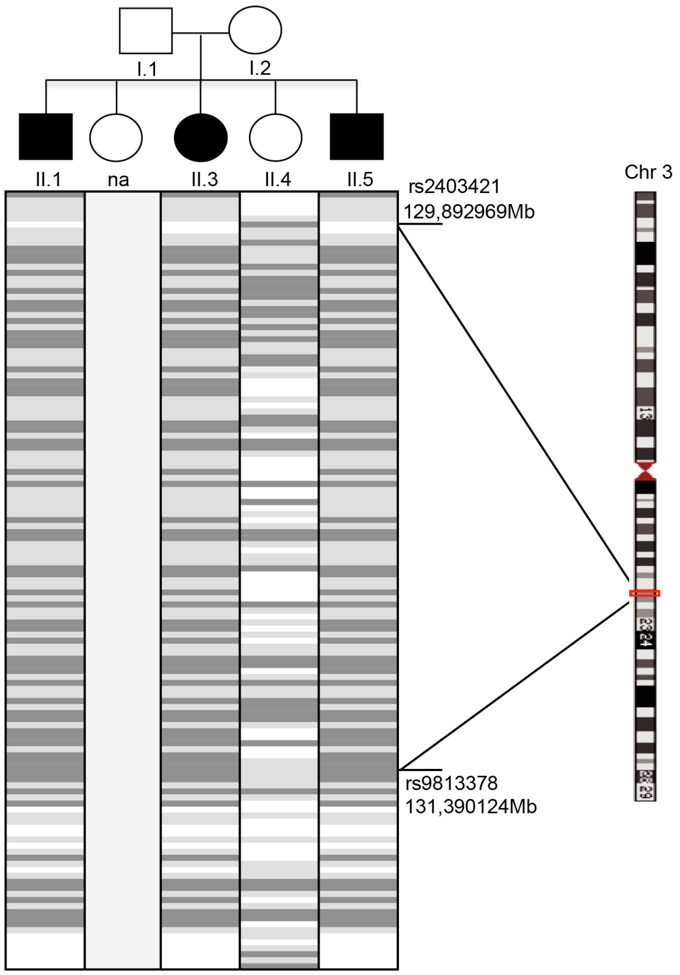

B

Chromosome	250K Affymetrix Array	genomic position (hg19)
Chr 3	rs2403421-rs9813378	129,892969 to 131,390124 Mb
	rs1907861-rs2126806	163,606756 to 164,286477 Mb
Chr 6	rs10944288-rs13202853	87,651742 to 88,505654 Mb
Chr 20	rs6032171-rs6104453	44,044003 to 44,712840 Mb

Supplementary Figure 1: Pedigree of the family with the homozygosity mapping. A. Pedigree of the family, underlined by corresponding schematic representation of the homozygosity mapping results that has been performed on 3 affected individuals and 1 unaffected individual using the Affymetrix 250K SNP array. This representation shows the chromosome 3 homozygous region that is common for the three affected individuals and the position of the *PIK3R4* gene. Gray shading indicates homozygous SNPs (light gray for AA and darker for BB) and white zones indicate heterozygous alleles (AB). **B.** Supplemental homozygous regions specific to the three affected individuals. Positions are given following GRCh37 hg19 reference genome.

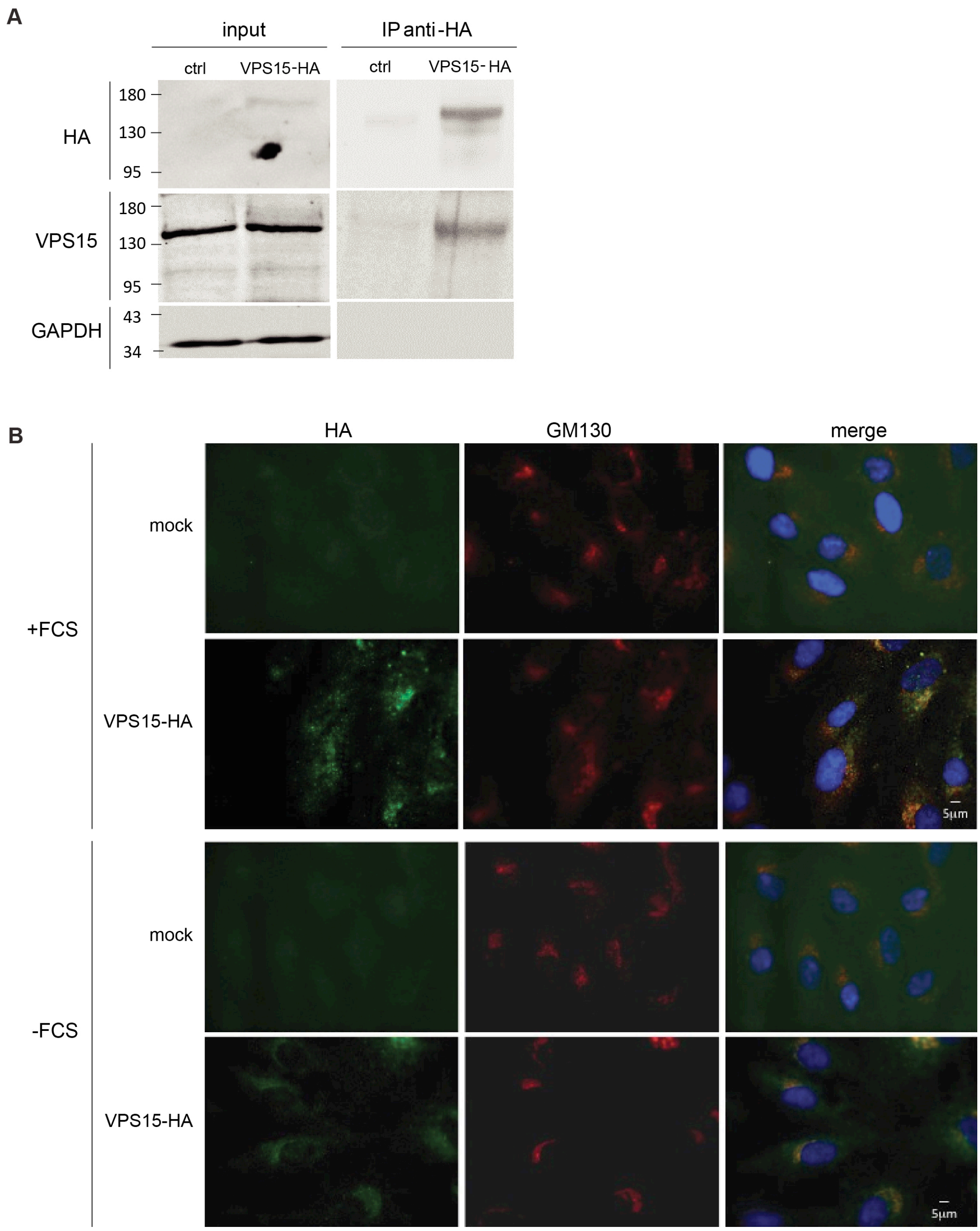

Supplementary figure 2: VPS15-HA expression from the VPS15-HA construction in hTERT-RPE1 cells and primary fibroblasts. A: hTERT-RPE1 cells were mock transfected (ctrl) or transfected with the VPS15-HA construct. 24h post transfection, cells were collected and an immunoprecipitation against HA performed. In WB HA was detected with an anti-HA antibody and VPS15 with the antibody directed against the WD40 region of VPS15. GAPDH was done as a control. **B:** Ctrl skin fibroblasts were either mock transfected or transfected with the VPS15-HA construct. 6h post transfection medium was changed and cells were either grown in complete medium (+FCS) or deprived of serum (-FCS) for 24h. Then DAPI staining as well as IFs against the VPS15-HA protein and the Golgi marker GM130 were performed.

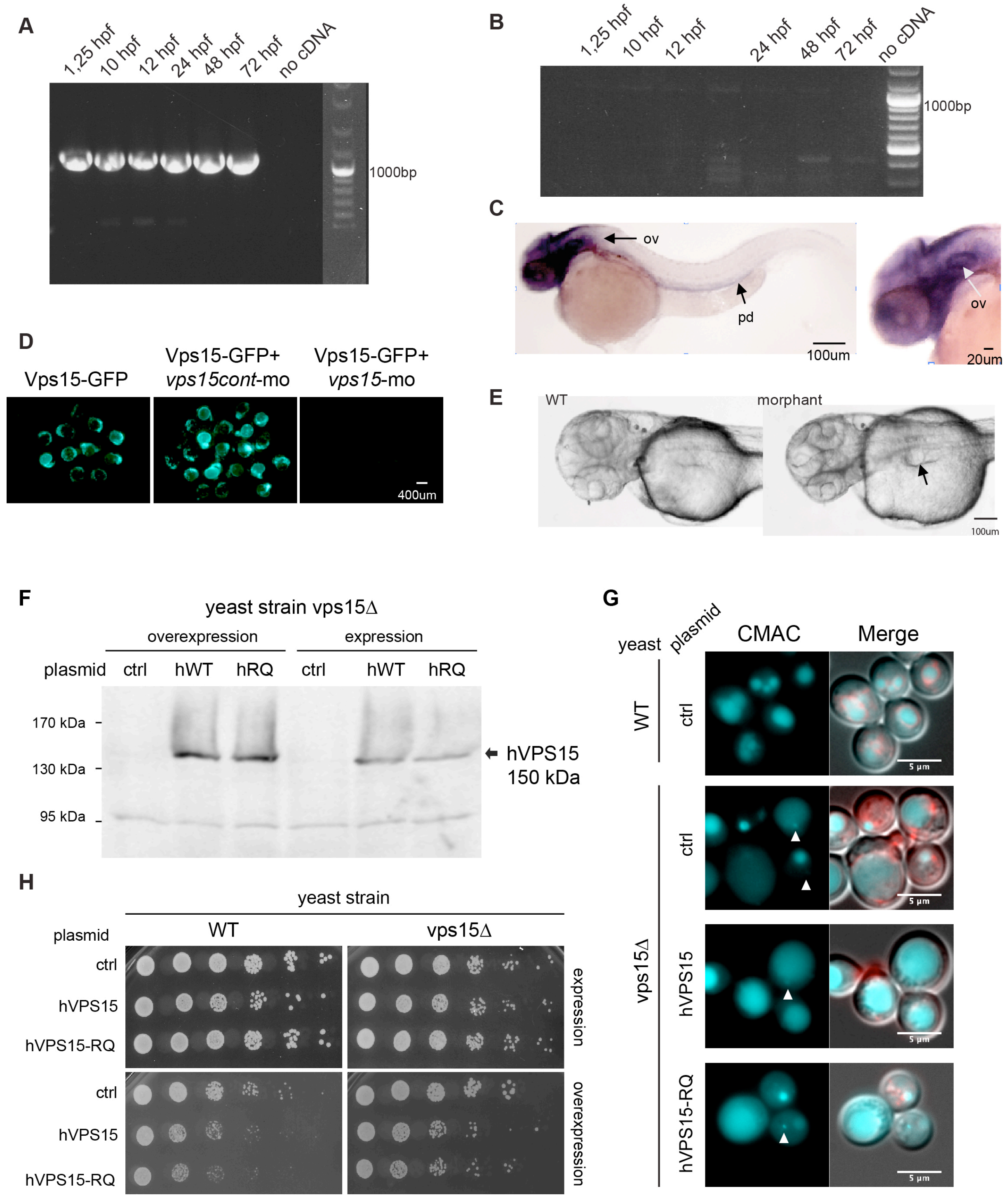

Supplementary Figure 3: Controls of zebrafish and yeast experiments. A-B: RT-PCR with cDNA of 6 different developmental stages (from 1,25 to 72 hpf) amplifies a 1000 bp band (arrow) as expected. *vps15* mRNA is maternally expressed before midblastula transition (1,25 hpf) and mRNA is detectable until at least 72 hpf. PCR without prior reverse transcription did not yield a 1000 bp band (**B**) indicating that the amplified fragment is not due to contamination with cDNA or genomic DNA**. C**: *In situ* hybridization with *vps15* antisense probe in zebrafish embryos showing ubiquitous expression at 14 hpf and prominent localization in the head as well as in the pronephric duct (pd) at 48 hpf. Ov: otic vesicle.

D. Co-injection of *vps15*-mo with zVps15-GFP plasmid completely blocked GFP expression. As expected, co-injection of the mismatch control *vps15cont*-mo with the zVps15-GFP reporter did not reduce the GFP expression as compared to controls injected with the zVps15-GFP plasmid. Embryos are 20 hpf old. **E** picture of WT and morphant zebrafish showing kidney cysts in the morphant (black arrow) but not the WT. **F.** *vps15*Δ yeast cells were transformed with a control empty plasmid (ctrl), an expression (CEN) or overexpression (2μ) plasmid bearing wild-type hVPS15 or mutant hVPS15-R998Q and a western blot against hVPS15 was performed. **G.** Wild-type (WT) and *vps15*Δ yeast cells transformed with a control (ctrl), hVPS15 or hVPS15-R998Q expression plasmid (CEN) were labeled with a soluble dye (CMAC) staining the lumen of the vacuole. White arrows show the additional CMAC positive compartment (CPC) in *vps15*Δ yeast cells. **H.** Drop test assays of wild-type WT and *vps15*Δ yeast cells expressing (CEN) or overexpressing (2μ) hVPS15 or hVPS15-R998Q. Plates were incubated at 30°C. Yeast strains transformed with an empty plasmid (ctrl) were used as controls.

A

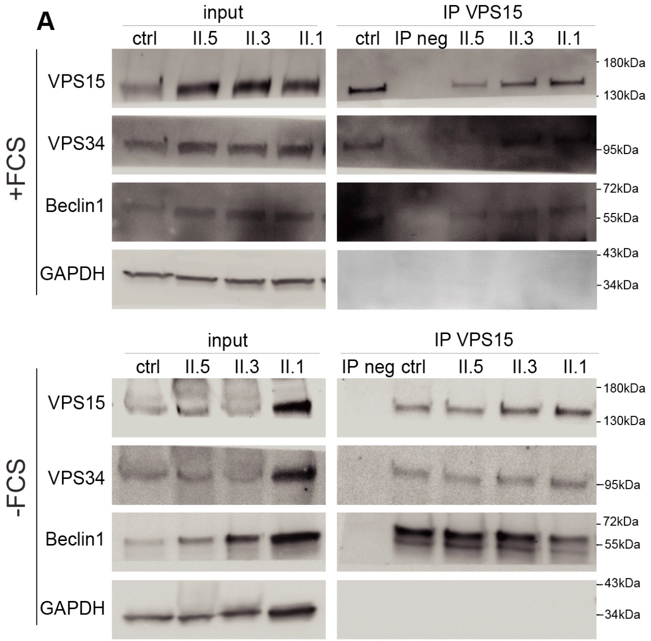

B

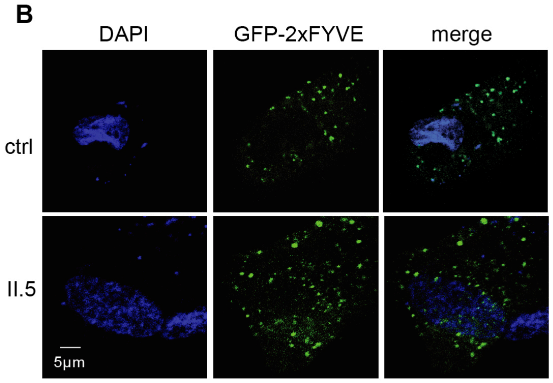

C

Protein	ACCESSION Nb	MW(kDa)	pI	Gene ID	mean nb spectra IPneg #Spectra	mean nb spectra IP #Spectra
VPS15	PI3R4_HUMAN	153.0	6.8	30849	0	140,8
VPS34	PK3C3_HUMAN	101.5	6.4	5289	0	93,6
GM130	GOGA2_HUMAN	113.0	4.9	2801	0	39,2
UVRAG	UVRAG_HUMAN	78.1	9.4	7405	0	31,8
Beclin1	BECN1_HUMAN	51.9	4.7	8678	0	31,4
Nrbf2	NRBF2_HUMAN	32.4	5.5	29982	0	21,6
Atg14L	BAKOR_HUMAN	55.3	6.7	22863	0	18,6

Protein	ctrl 1 sample 26032015 IP-VPS15_ctrl1					II.5 sample 26032015 IP-VPS15_2-5				
	Score	#Peps	#Spectra	SC(%)	RMS(ppm)	Score	#Peps	#Spectra	SC(%)	RMS(ppm)
VPS15	1787	32	84	30	4,02	2595	40	112	37	7,82
VPS34	1115	18	43	23,8	3,98	1432	20	68	29,2	8,3
GM130	719,6	12	23	18	10,33	1035	14	31	21,3	9,79
UVRAG	475	8	14	14,2	4,17	505,6	8	16	14,4	6,61
Beclin1	484,6	7	17	22	4,04	571,6	7	18	21,1	6,71
Nrbf2	416,5	7	10	27,9	5,84	366,5	5	10	34,1	5,02
Atg14L	390,7	5	11	16,3	3,63	501,7	7	15	22,6	4,8

Protein	ctrl 2 sample 09062015 IP-VPS15_ctrl2					II.1 sample 09062015 IP-VPS15_2-1					II.3 sample 09062015 IP-VPS15_2-3				
	Score	#Peps	#Spectra	SC(%)	RMS(ppm)	Score	#Peps	#Spectra	SC(%)	RMS(ppm)	Score	#Peps	#Spectra	SC(%)	RMS(ppm)
VPS15	3674	56	208	45,8	5,48	3880	58	163	48,5	6,55	3512	52	137	46,4	5,47
VPS34	2962	46	155	58,5	6,36	3096	44	121	59,9	8	2172	32	81	41,4	5,15
GM130	1465	21	49	30,3	4,1	1593	22	51	33,4	5,47	1632	20	42	31,1	4,04
UVRAG	1336	25	56	38,6	6,41	1218	20	37	36,2	5	1138	20	36	39,3	4,11
Beclin1	1223	19	56	40,7	4,17	1162	17	33	37,8	6,61	1136	18	33	47,1	5,09
Nrbf2	585	7	33	41,8	9,28	687,4	12	32	64,1	11,78	524,8	8	23	40,4	5,69
Atg14L	666	10	32	28,7	3,63	654	8	19	25,8	4,72	525,1	6	16	19,9	2,08

Supplementary Figure 4: VPS15 and VPS15-R998Q protein complexes and PtdIns(3)P staining in human fibroblasts. A. Control (ctrl) and patient (II.1, II.3 and II.5) fibroblasts were grown in complete medium (+FCS) or deprived of FCS for 24 hours. Immunoprecipitation with a VPS15 antibody was done on cell lysates (input) and interaction (IP VPS15) with the indicated proteins (VPS34, Beclin1 and GAPDH) was detected by western blot. IPneg represent the control immunoprecipitation done without VPS15 antibodies. **B.** Control and patient II.5 fibroblasts were grown in complete medium and transfected with a GFP-2XFYVE plasmid, 24 hrs post transfection cells were observed on a fluorescence microscope. **C.** VPS15 was immunoprecipitated from control (ctrl1 and ctrl2) and patient (II.5, II.1 and II.3) fibroblast protein lysates and samples prepared for and analysed by mass spectrometry. Interaction partners were determined by searching against the complete Human proteome set from the SwissProt database. All retained interaction partners were completely absent from the negative control immunoprecipitations (IPneg) omitting VPS15 antibodies (mean number of spectra “0” in the IPneg column) but present in all five VPS15 immunoprecipitation samples (ctrl1, ctrl2, II.5, II.1 and II.3).

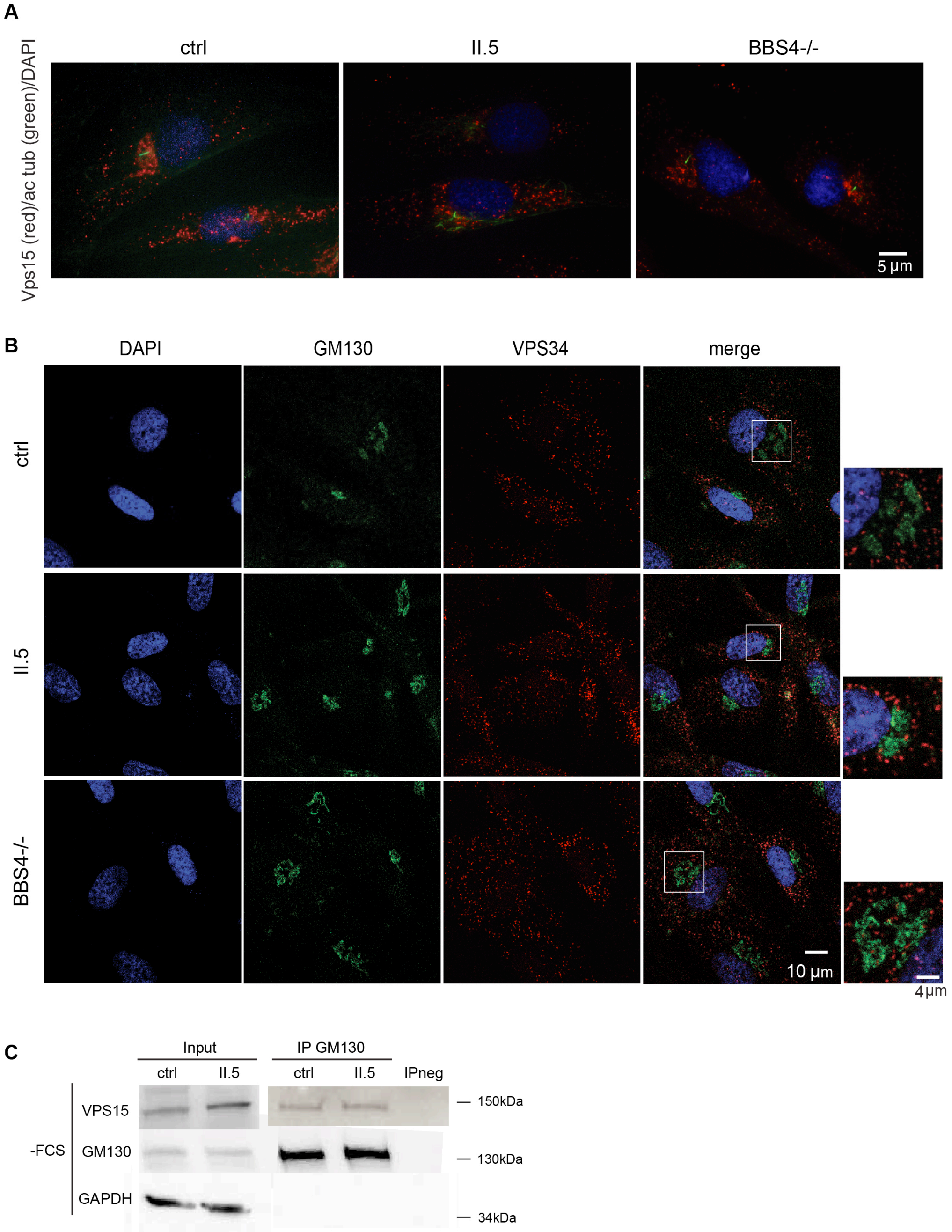

Supplementary Figure 5: Primary cilia and VPS15 do not colocalize and VPS34 is not Golgi-localized. A. Control (ctrl) and patient II.5 fibroblasts were deprived of serum for 24 hrs and fixed. Immunofluorescence against acetylated tubulin (green) and VPS15 (red) and DAPI staining were performed and pictures taken on a fluorescence microscope, only the merge is shown. **B.** Control (ctrl) and patient II.5 fibroblasts were deprived of FCS for 24 hrs and fixed. Immunofluorescence against GM130 (green) and VPS34 (red) and DAPI (blue) staining were performed and pictures taken on a confocal microscope. White square: zoomed area. **C.** Control (ctrl) and patient (II.5) fibroblasts were deprived of serum for 24 hrs. An immunoprecipitation against GM130 was performed on total cell lysates and VPS15 and GM130 detected by western blot. Negative immunoprecipitation without antibodies (IP neg) was used as a control.

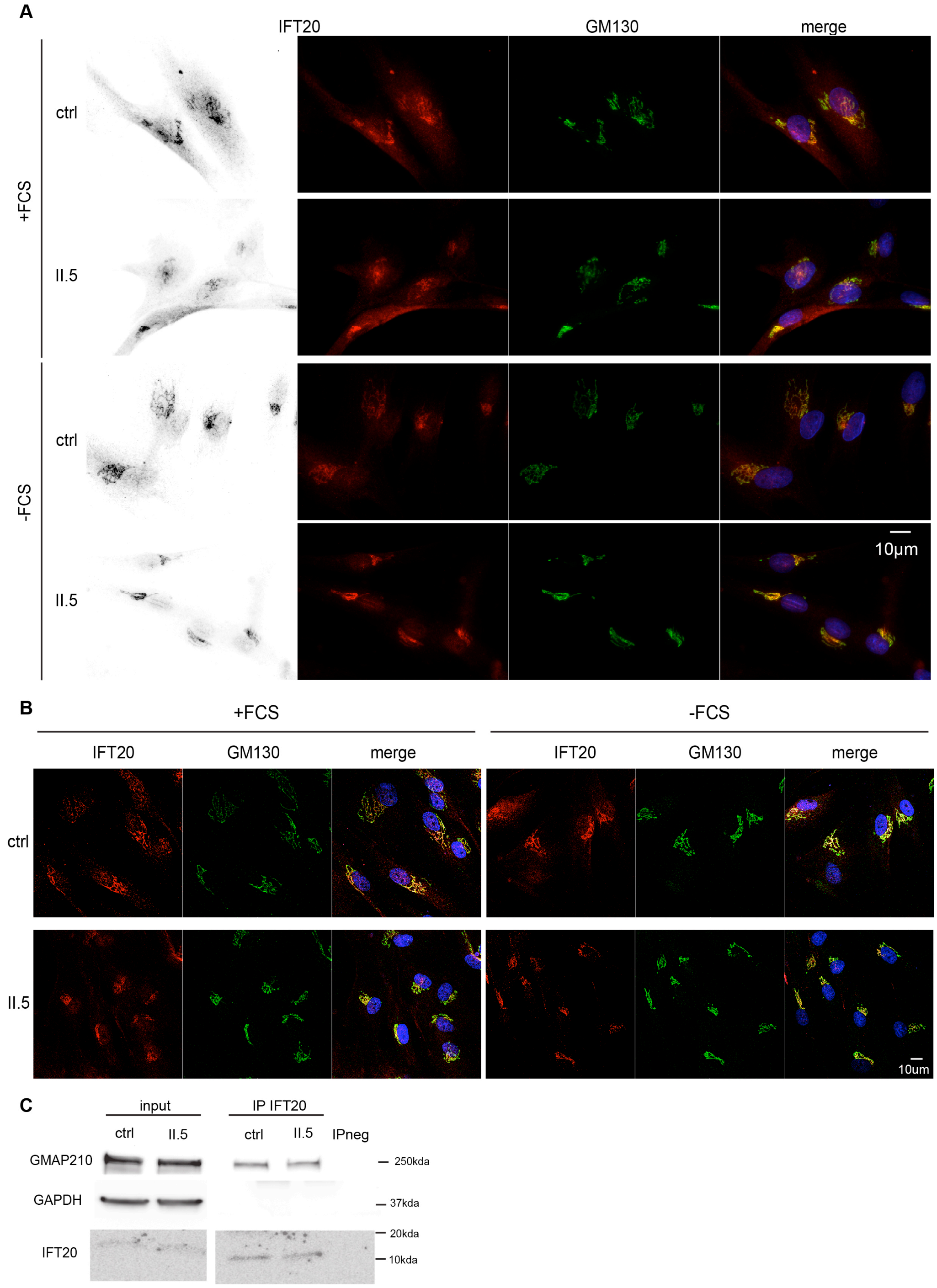

Supplementary Figure 6: Intracellular localization of IFT20 in control and patient fibroblasts. A. Control (ctrl) and patient II.5 fibroblasts were grown in complete medium (+FCS) or deprived of serum for 24 hrs (-FCS) and fixed. Immunofluorescence against IFT20 (red and inverted black/white panel) and GM130 (green), and DAPI staining were performed and localization of IFT20 in the cells was observed on a fluorescence microscope. **B.** The same immunofluorescence stained slides were observed with a confocal microscope. **C.** Control (ctrl) and patient (II.5) fibroblasts were deprived of serum for 24 hrs. An immunoprecipitation against IFT20 was performed on total cell lysates and GMAP210 and IFT20 detected by western blot. Negative immunoprecipitation without antibodies (IP neg) was used as a control.

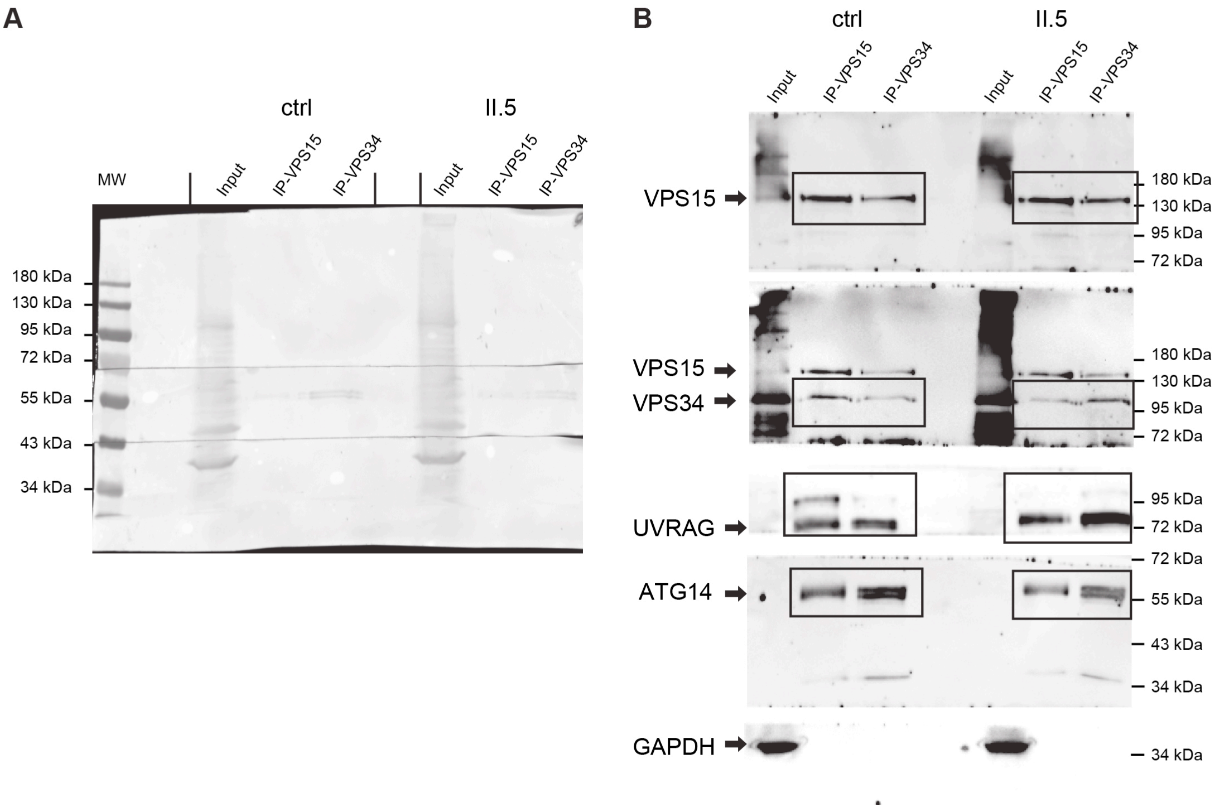

Supplementary Figure 7: Uncropped images of western blots for Figure 4B. A. Ponceau staining of the blot, the blot was cut in several pieces that were incubated with the indicated antibodies. **B.** Western blots were revealed with anti-VPS34, -VPS15, -UVRAG, -ATG14 and -GAPDH antibodies, as indicated.

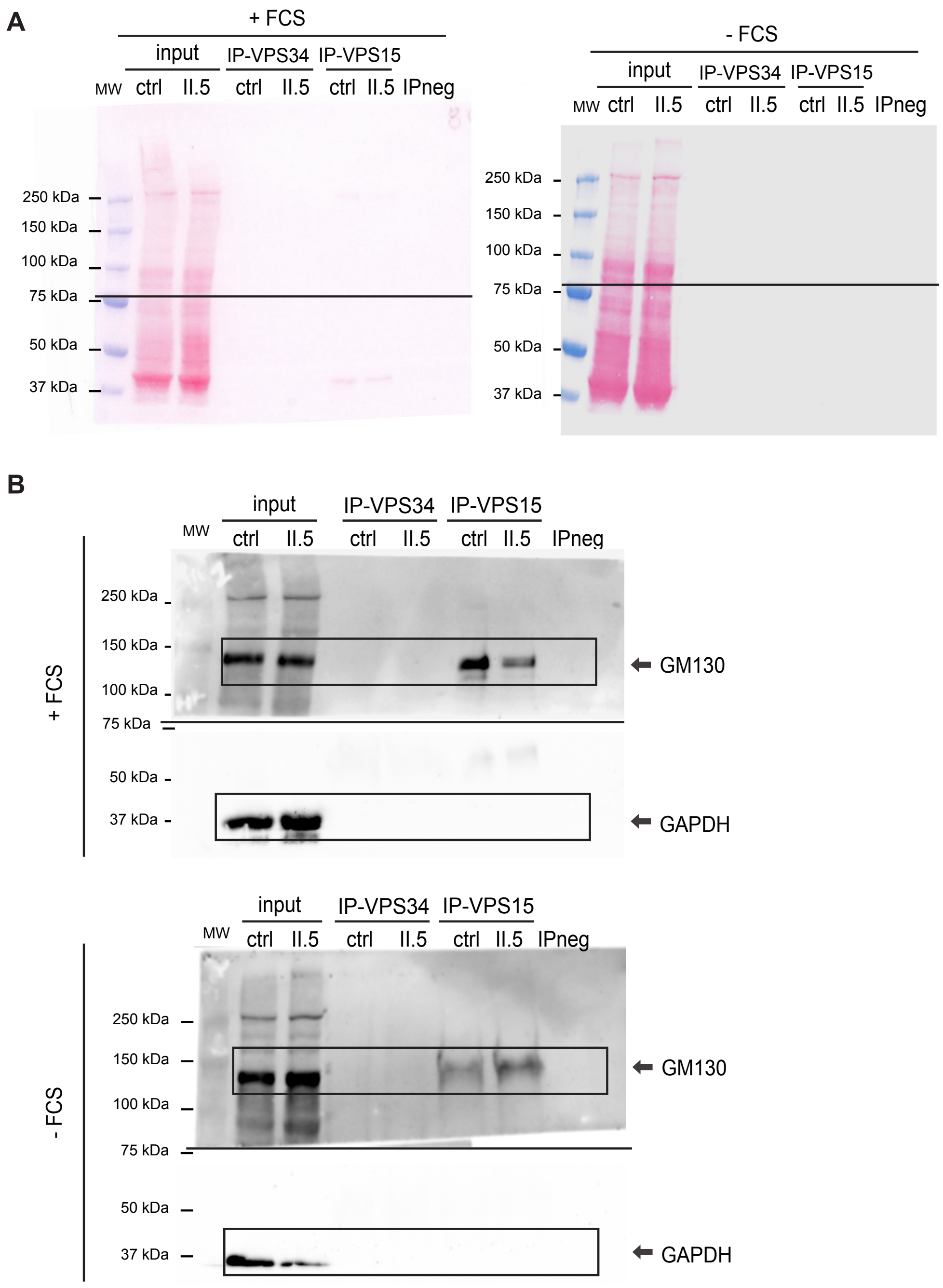

Supplementary Figure 8: Uncropped images for western blots for Figure 5A. A. Ponceau staining of the two blots, the blots were cut in two pieces that were incubated with the indicated antibodies. **B.** Western blots were revealed with anti-GM130 and anti-GAPDH antibodies, as indicated.

Patients	II.1		II.3		II.5		II.4	
**Type of sequence variant**	SNV	Indel	SNV	Indel	SNV	Indel	SNV	Indel
Total number of variants	52646	8185	52434	8178	52576	8253	55365	8522
After exclusion of non-pathogenic variants (as determined from the ClinicalSignificance field in dbSNP) validated by at least 2 methods in dbSNP (as determined from the "Validation Status" field)	8980	5296	8427	5294	8962	5378	10899	5578
After exclusion of variants with an allele frequency > 1% (extracted from the dbSNP database, the Exome Variant Server, the 1000g and the ExAC database)	7132	3790	6533	3813	7081	3883	8830	4084
After exclusion of variants found in the homozygous state + exclusion of variants found more than once in the heterozygous state in 70 control exomes	2193	844	1633	816	2199	892	4227	926
After exclusion of 5'UTR, 3'UTR, downstream, upstream and intron locations without local splice effect prediction (from the "localSpliceEffect" field of Alamut-Batch)	1120	204	778	196	1121	233	2190	245
After exclusion of synonymous variants without local splice effect prediction (from the "localSpliceEffect" field of Alamut-Batch)	881	204	597	196	891	233	1764	245
After selection of variants consistent with recessive transmission (compound heterozygous, homozygous variants).	1 homozygous variant in the *PIK3R4* gene<br>2 compound heterozygous variants in the *AK9* and *RECQL4/MFSD3* genes							

Supplementary Table 1: Summary of the exome sequencing results from the three affected probands and the healthy sister.

RefSeq Gene	Gene	Forward (5'-3')	Reverse (5'-3')
Human VPS15/PIK3R4 NM_014602.2	PIK3R4-ex2	TGGTGTTTCACAGCTTCTTTG	GCAGGAATGCTGCCTCTAAC
	PIK3R4-ex3	GGTTTTGCATTTTGGGTTTT	TTAAAGCAAAAGTGGGAACTAGA
	PIK3R4-ex4	GCCTTTGTAGCTTTCATCTGG	TGAGAGAACACACAAACGTTGA
	PIK3R4-ex5	GACAGGGAGGAAAAGTTAATAAGC	GTTTTACAAGTTAGCATCAGCAATTC
	PIK3R4-ex6	TGGGACTATCTGTATGGGGAAA	TGCATGGAAGTATTTGAGAGGA
	PIK3R4-ex7	TCCTTACATGTCTTGAAATGACTTT	AGTAGGAACCCCACACAATCA
	PIK3R4-ex8	TTTGTTTATCCCGATAGTTTGAA	CCTTCAAAGGTGCTTGGTTC
	PIK3R4-ex9	CTTCTACACACTTACAGATGTTG	TGCCTAAGATGTTTGCCAGA
	PIK3R4-ex10	TGGGTGATTTGTCCCTTCAG	TCTGTGAAATGGGGCTTTTT
	PIK3R4-ex11	GGCAATAAAAGCACATAATGGTT	CGTTCAAAGACATTAGGAGGAC
	PIK3R4-ex12	TGTCTTCAAATATACTTTGCTTACGAC	TTCTGGGTTTCCCAAATAACA
	PIK3R4-ex13	TTGCCAAATTGATGTCTCAG	CACAGAAGAAGGATTTTTGTCCA
	PIK3R4-ex14	TTTAACATTAGCAGTGACATTGGTT	CATTGCAGGGTAGAAAGGAGA
	PIK3R4-ex15	TCGGCTTTCGTTTTCTTGAT	CAGGCATTAGTGGCATTCAA
	PIK3R4-ex16	ATGCAGCTTGATGCACTTTG	CCAAGAAAATCTCCCAACACA
	PIK3R4-ex17-ex18	TGTCTCTCCCCCTGAAAGAA	TGATTTTAAACATTACAGTGCCATC
	PIK3R4-ex18-ex19	CGGATGTCTTTTATTTGACTCCA	CACTTTCTTTATGCTTATTTTAC
	PIK3R4-ex20	CTGACGAGAGGAAAAATGTG	CCACCAAAACCACAAACTCTT
Zebrafish *zvps15/pik3r4* ZDB-GENE-050309-84	pikwt	cttgaattcATGGGGAACCAACT GGCTGGAATTGCTCCATCG	cttctcgaggggACTTCCAGACTTT GACAATGCCATCTCTAGAG
	pikmut	GCACAAATCTGCTGTCAAC CaAATCCGAGTCTCAGATGAG	CTCATCTGAGACTCGGA TTtGGTTGACAGCAGATTTGTGC
	pik3r4WM	gtgggagaagcatctgtgatc	ctacacaggttgttgaggatg

Supplementary Table 2: Primers used in this study

List of antibodies used in this study			
**Name Primary Antibodies**	**Applications**	**Producer**	**Conditions**
Mouse Anti-Acetylated alpha Tubulin [6-B11-1]	western, IF	Abcam #ab24610	IF: 1/200
Rabbit Anti-PIK3R4	western, IF, IP	Novus Biologicals #NBP1-30463	WB 1/1000 IF: 1/200-1/300 IP: 1µl Ab/100µg prot
Mouse Anti-PIK3R4	IF	Abnova #H00030849-M02	IF:1/100
Rabbit anti-Beclin-1	Western	Cell Signaling #3495	WB: 1/1000
Anti-UVRAG	Western	Abcam #ab174550	WB: 1/1000
Rabbit Anti-GAPDH	Western	Abcam #ab181602	WB: 1/5000
Rabbit Anti-PI3 Kinase Class 3 (VPS34)	IF	Abcam #ab137784	IF : 1/500
Mouse Anti-PI3 Kinase Class 3 (D9A5) (VPS34)	IP, Western, IP	Cell Signaling #5289	WB: 1/1000 IP: 200µg Prot+ 2µl Ab
Mouse Anti-GM130	Western, IF	Abcam #ab169276	western: 1/1000 IF:1/250 IP: 2µl/200µg Prot
Rabbit Anti-IFT20	Western, IF, IP	Proteintech #13615-1-AP	WB:1/1000-2000 IF: 1/300 IP:300µg Prot + 3µl Ab
RabbitAnti-Atg14	Western	Cell Signaling #5504	WB:1/1000
Mouse Anti- GMAP210	Western	Thermo Scientific #MA1-23294	WB:1/500
Mouse anti-HA	Western, IF	Abcam #ab130275	WB: 1/1000 IF: 1/200
Rat monoclonal Anti-HA	Western , IF	Roche #1 867 423	WB:1/1000 IF:1/200
**Name Secondary Antibodies**	**Applications**	**Producer**	**Conditions**
Mouse Anti-rabbit IgG (Conformation Specific) (L27A9) mAb (HRP Conjugate)	Western	Cell Signaling #5127	WB:1/1000
Donkey anti rabbit IgG FITC	IF	Santa Cruz #sc-2090	IF:1/200
Donkey anti-Mouse IgG (H+L), Alexa Fluor 594 conjugate	IF	Thermo Scientific A-21203	IF:1/500
Chicken anti-Rabbbit IgG-HRP	Western	Santa Cruz #sc-2955	WB: 1/5000
Goat anti-mouse Alexa Fluor coupled (either 488 or 568) IgG (Invitrogen)	IF	Thermo Scientific #A-11001 #A-11031	IF: 1/500
Goat anti-Rabbit IgG (H+L) Alexa Fluor 568 conjugate	IF	Thermo Scientific # A-11011	IF:1/400
Goat anti-rat Alexa Fluor 488 coupled IgG	IF	Abcam #ab150157	IF:1/600
Donkey anti-Mouse IgG (H+L), FITC conjugate	IF	Thermo Scientific #A16012	IF:1/500
Goat anti mouse IgG-HRP	Western	Santa Cruz #sc-2060	WB: 1/5000
Goat anti mouse IgG FITC	IF	Thermo Scientific # A16085	IF:1/400

Supplementary Table 3: Antibodies used in this study
